# Mitochondrial retrograde signal through GCN5L1 transition–mediated PPAR**γ** stabilization promotes MASLD development

**DOI:** 10.1172/jci.insight.196695

**Published:** 2026-01-23

**Authors:** Jiaqi Zhang, Danni Wang, Qiqi Tang, Yaoshu Yue, Xin Lu, Xiuya Hu, Yitong Han, Jiarun Chen, Zihan Wang, Xue Bai, Kai Zhang, Yongsheng Chang, Longhao Sun, Lu Zhu, Lingdi Wang

**Affiliations:** 1 Department of Physiology and Pathophysiology, State Key Laboratory of Experimental Hematology, Tianjin Key Laboratory of Inflammatory Biology, The Province and Ministry Co-sponsored Collaborative Innovation Center for Medical Epigenetics, NHC Key Laboratory of Hormones and Development, Chu Hsien-I Memorial Hospital and Tianjin Institute of Endocrinology, School of Basic Medical Sciences, Tianjin Medical University, Tianjin, China.; 2 Department of Pharmacology, School of Basic Medical Sciences, Tianjin Medical University, Tianjin, China.; 3 Department of General Surgery, Tianjin Medical University General Hospital, Tianjin, China.; 4 Department of Biochemistry and Molecular Biology, School of Basic Medical Sciences, Tianjin Medical University, Tianjin, China.

**Keywords:** Cell biology, Hepatology, Mitochondria, Signal transduction

## Abstract

Mitochondrial retrograde signaling plays crucial roles in maintaining metabolic homeostasis via regulating genome modification and oxidative responsive gene expression. In this study, we identified GCN5L1, a protein localized in both mitochondria and cytoplasm, and demonstrated its specific translocation from mitochondria to cytoplasm during lipid overload and high-fat diet feeding. Using transcriptome and proteome analyses, we identified that cytoplasmic GCN5L1 binds to and promotes the acetylation of PPARγ at lysine 289 (K289). This acetylation protected PPARγ from ubiquitination-mediated degradation by proteasome. GCN5L1 translocation enhanced protein stability of PPARγ and subsequently promoted lipid accumulation in both cultured cells and murine models. Our study further reveals that PPARγ-K289 mutation reduces the ubiquitination of PPARγ and exacerbates liver steatosis in mice. These findings unveil a mitochondrial retrograde signaling during lipid overload, which regulates the crucial lipogenic transcriptional factor. This discovery elucidates an unrecognized mitochondrial function and mechanism underlying hepatic lipid synthesis.

## Abstract 2

Mitochondrial GCN5L1 translocates to the cytoplasm and modulates acetylation at PPARγ-K289 to protect it from degradation, thereby promoting HFD induced liver steatosis.

## Graphical abstract

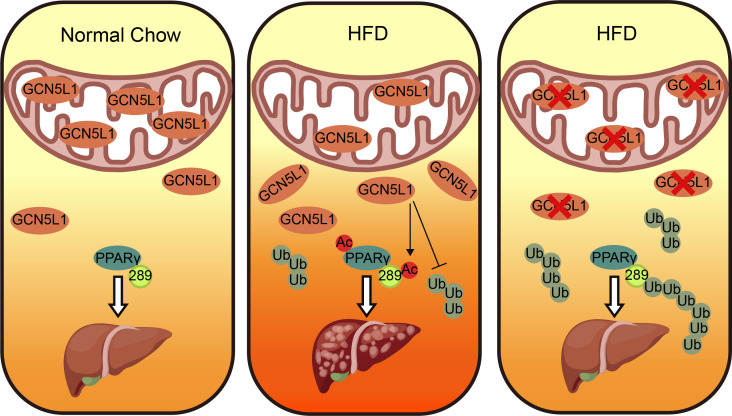

## Introduction

Mitochondria serve as central regulators of hepatic lipid metabolism, mediating fatty acid oxidation (FAO) and supplying TCA cycle metabolic precursors (e.g., citrate) for lipid biosynthesis via cytoplasmic acetyl-CoA export (1, 2). The balance between mitochondrial FAO and lipogenic precursor supply is pivotal for metabolic homeostasis, and its dysregulation is a key pathophysiological mechanism of metabolic dysfunction–associated steatotic liver disease (MASLD). Therefore, therapeutic strategies targeting mitochondrial FAO have gained clinical interest; however, currently no drug has been approved by the FDA that solely targets FAO. The FDA-approved drug resmetirom ameliorates metabolic dysfunction–associated steatohepatitis (MASH) through dual modulation of mitochondrial FAO and inflammatory pathway suppression to reduce liver steatosis, liver injury, and fibrosis (3). For simple liver steatosis, exercise remains first-line for lipid reduction, partly as excessive FAO may induce oxidative stress, exacerbating mitochondrial stress, reactive oxygen species–mediated (ROS-mediated) hepatic injury, and lipid metabolism disruption (4). Mitochondrial dysfunction is linked to MASLD, evidenced by damaged mitochondrial structure from TEM and altered mitochondrial respiration (5, 6). Therefore, maintaining proper mitochondrial function is crucial for preventing excessive lipid accumulation and protecting hepatocytes from oxidative stress. Notably, the role of mitochondria in hepatic lipid synthesis remains poorly understood. More evidence regarding the function of liver mitochondria in lipid metabolism would uncover new therapeutic potential for maintaining the homeostasis of hepatic lipid metabolism.

Mitochondria are recognized as the powerhouse of the cell, and their emerging role as signaling hubs has gained substantial attention. To date, mitochondrial signaling encompasses multiple components, including ROS, mitochondrial released metabolites, mitochondrial membrane potential, and mitochondrial protein translocation (7, 8). However, the specific signals involved in the regulation of lipid synthesis remain largely elusive. Mitochondrial retrograde signaling is complex, e.g., ROS activates antioxidant responses, calcium signaling contributes to various cellular processes, and the AMP/ATP ratio activates AMPK for energy homeostasis. Regarding protein translocation, multiple studies have observed this phenomenon, particularly in the context of the mitochondrial integrity stress response, which induces mitochondrial proteins to change their mitochondrial localization, such as ATF5, DELE1, and SSBP1 (9–11). Mitochondria contain approximately 1,200 proteins, of which 60%–70% possess a mitochondrial targeting sequence (MTS), which determines their localization in mitochondria (12). Recent research has demonstrated that, in response to mitochondrial dysfunction, the proteinase OMA1 located in mitochondrial intermembrane space processes proteins with an MTS, thereby altering the mitochondrial proteins (10). In contrast, for proteins lacking an MTS, it remains largely unknown whether they could translocate to a new localization to acquire new functions, and if so, what the underlying mechanisms are.

GCN5L1, also referred to as BLOC1S1, is a component of the BLOC1 complex. Previous studies have identified that GCN5L1 exhibits dual localization: within the mitochondria, it functions as a protein acetylation regulator; in the cytoplasm, it acts as an adaptor and acetylation regulator that links the lysosome and tubulin, mediating lysosome trafficking, a process that depends on tubulin acetylation (13). However, our prior research has demonstrated that in the liver, GCN5L1 has a predominantly mitochondrial localization (14, 15). This localization is associated with mitochondrial protein acetylation and metabolic alteration to modulate FAO in response to high-fat diet (HFD) feeding in a mouse model (16). Interestingly, emerging evidence indicates that GCN5L1 is also present on lysosome membrane, where it modulates FAO via lysosome-dependent metabolic pathway (17, 18).

In the current study, we aimed to explore the specific compartmentation of GCN5L1 in the regulation of hepatic lipid metabolism. We discovered that GCN5L1/BLOC1S1 undergoes a significant translocation during MASLD development. In both a mouse model and human MASLD patients, mitochondria-localized GCN5L1 decreases substantially, while cytoplasmic GCN5L1 accumulates. Concurrently with the relocation of GCN5L1, protein acetylation in both mitochondria and cytoplasm changed in accordance with the new localization of GCN5L1 during MASLD progression. Specifically, cytoplasmic GCN5L1 binds to peroxisome proliferator–activated receptor γ (PPARγ) and acetylates it at the lysine 289 (K289) residue. This acetylation competitively inhibits the ubiquitination of PPARγ at this residue, thereby protecting PPARγ from proteasome-mediated degradation. These findings elucidate the mechanism by which mitochondrial retrograde signaling regulates hepatic de novo lipogenesis via the cytoplasmic GCN5L1/PPARγ axis during MASLD progression. As such, this axis may represent a potential target and a new aspect for the prevention and treatment of MASLD.

## Results

### GCN5L1 accumulates in cytoplasm during the development of MASLD in both HFD-fed mice and MASLD patients.

**Figure 1:**
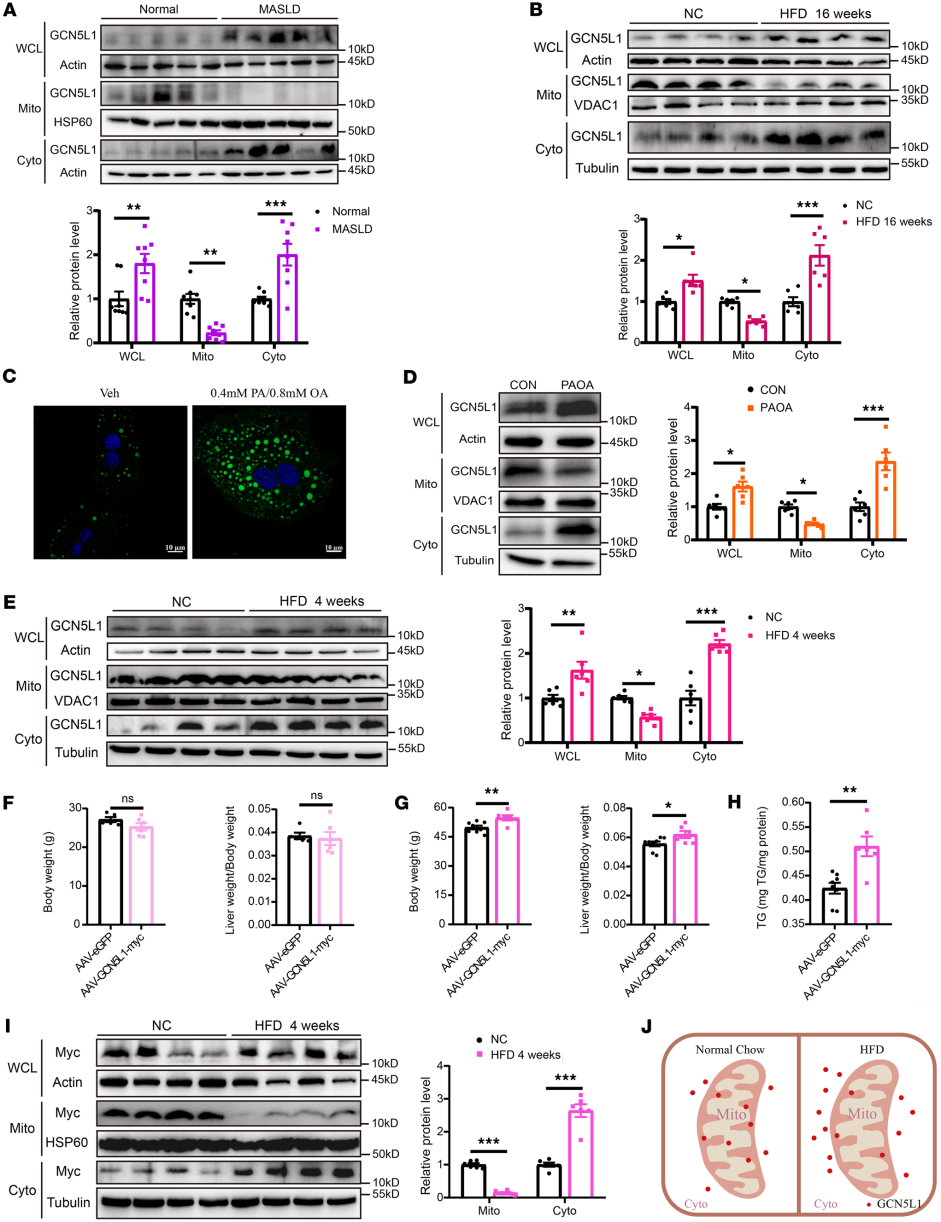
GCN5L1 accumulates in cytoplasm during development of MASLD in both HFD-fed mice and MASLD patients. (**A**) GCN5L1 protein levels and quantification of whole-cell lysate (WCL), mitochondrial (Mito), and cytoplasmic (Cyto) fractions of liver biopsies from healthy control (normal) or MASLD patients. *n* = 8 for each group. (**B**) GCN5L1 protein levels and quantification of WCL, Mito, and Cyto fractions of liver samples from mice fed normal chow (NC) or 16 weeks of high-fat diet (HFD). *n* = 6 mice per group. (**C**) Representative immunofluorescence of BODIPY and DAPI in primary hepatocytes with or without 0.4 mM PA/0.8 mM OA treatment overnight. (**D**) GCN5L1 protein levels and quantification of WCL, Mito, and Cyto fractions of primary hepatocytes treated with or without 0.4 mM PA/0.8 mM OA treatment overnight. Quantitation of 6 independent experiments is shown. (**E**) GCN5L1 protein levels and quantification of WCL, Mito, and Cyto fractions of liver samples from mice fed NC or 4 weeks of HFD. *n* = 6 mice per group. (**F**–**H**) Wild-type mice were injected with AAV-eGFP or AAV-GCN5L1-myc via tail vein and subjected to NC or HFD for 16 weeks. Body weight, liver/body weight ratio (**F** and **G**), and liver triglyceride content (**H**) were assessed. *n* = 5 for AAV-eGFP with NC, *n* = 8 for AAV-eGFP with HFD, *n* = 6 for AAV-GCN5L1-myc. (**I**) GCN5L1-myc protein levels and quantification of WCL, Mito, and Cyto fractions of liver samples from mice fed NC or 4 weeks of HFD. *n* = 6 mice per group. (**J**) Illustration of GCN5L1 protein distribution during MASLD or fatty acid incubation. Datasets in **A**, **B**, **D**, **E**, and **I** were analyzed by 2-way ANOVA with Bonferroni correction. **F**–**H** datasets were analyzed by 2-tailed Student’s *t* test. ns, *P* > 0.05; *P < 0.05, **P < 0.01, ***P < 0.001.

As GCN5L1 expression has been reported to be upregulated in an acute HFD feeding murine model (16), we sought to explore the role of GCN5L1 in the progression of MASLD in human patients and mouse models. Analysis of the Gene Expression Omnibus (GEO) database revealed elevated GCN5L1 expression in individuals with MASLD (also referred to as non-alcoholic fatty liver disease) (Supplemental Figure 1A; supplemental material available online with this article; https://doi.org/10.1172/jci.insight.196695DS1). Given that both mitochondrial and lysosomal GCN5L1 regulates hepatic lipid homeostasis through distinct mechanisms (16, 18), we hypothesized that the subcellular localization of GCN5L1 is crucial for maintaining hepatic lipid homeostasis during MASLD progression. To test this, we evaluated GCN5L1 protein levels and localization in human liver biopsies from 8 healthy individuals and 8 MASLD patients. Metabolic parameters of these individuals are compared in Supplemental Table 1 to confirm their disease status. Consistent with the GEO analysis, both transcriptional and protein levels of GCN5L1 in liver lysates were significantly increased in MASLD patients. Notably, we observed a marked reduction in mitochondrial GCN5L1 levels in MASLD patients compared with healthy controls, while cytoplasmic GCN5L1 levels increased substantially, contributing to the overall elevation in total GCN5L1 protein (Figure 1A). In line with these findings in humans, this phenotype was recapitulated in a mouse model of MASLD, comparing the livers of mice fed an HFD for 16 weeks with those of mice fed normal chow (NC) (Figure 1B). Since GCN5L1 is a regulator of mitochondrial protein acetylation, we examined mitochondrial protein acetylation in patient biopsy and murine MASLD liver samples. As expected, the levels of mitochondrial protein acetylation were significantly decreased in MASLD livers from patients and HFD-fed mice compared with controls, indicating that reduced mitochondrial GCN5L1 levels were associated with decreased mitochondrial protein acetylation during MASLD progression (Supplemental Figure 1, B and C).

To further validate our findings, we incubated palmitic acid and oleic acid (PA/OA) with primary mouse hepatocytes to mimic MASLD development (19). Lipid accumulation induced by PA/OA treatment was visualized by BODIPY staining (Figure 1C), and immunoblotting confirmed the translocation of GCN5L1 from mitochondria to the cytoplasm (Figure 1D), consistent with our in vivo observations. Together, the results demonstrate that during MASLD progression, increased GCN5L1 expression coincides with cytoplasmic GCN5L1 accumulation, while reduced mitochondrial GCN5L1 levels may be linked to enhanced FAO. These findings suggest that the accumulation of cytoplasmic GCN5L1 during MASLD associates with lipid accumulation, potentially through mechanisms independent of mitochondrial lipid metabolism regulation, such as FAO.

To validate the link between cytoplasmic GCN5L1 accumulation and MASLD progression, we examined the critical time of GCN5L1 translocation and hepatic lipid accumulation during HFD feeding. In mice fed an HFD for 2 weeks, liver mitochondrial and cytoplasmic GCN5L1 protein levels remained similar to those of mice fed normal chow diet (NCD), as did hepatic triglyceride (TG) levels (Supplemental Figure 1, D and E). However, after 4 weeks of HFD feeding, mitochondrial GCN5L1 levels decreased slightly, while cytoplasmic and whole-liver-lysate GCN5L1 levels rose significantly, in line with increased hepatic TG content (Figure 1E and Supplemental Figure 1E). Meanwhile, GCN5L1 transcription increased in livers of mice fed an HFD for 4 weeks and 16 weeks, as well as in primary hepatocytes with PA/OA treatment compared with vehicle treatment (Supplemental Figure 1F), suggesting that HFD-induced GCN5L1 transcription correlated with cytoplasmic GCN5L1 accumulation and hepatic TG buildup.

The increased mitochondrial localization of GCN5L1 is observed in livers with acute HFD feeding, which regulates the acetylation of HADHA to modulate its function, and FAO substantially impacts MASLD progression (16). MASLD is characterized by mitochondrial dysfunction, including decreased membrane potential and ATP synthesis defects, which activates mitochondrial integrated stress response (ISR) to affect mitochondrial protein localization, e.g., DELE1 (10, 20). Meanwhile, mitochondrial membrane potential is crucial for protein import into mitochondrial matrix (12). To identify factors driving GCN5L1 translocation, especially decreased mitochondrial localized GCN5L1, two potential explanations were considered: reduced mitochondrial import of GCN5L1, and the release of GCN5L1 from mitochondrial matrix to cytoplasm. We first treated primary hepatocytes with oligomycin or FCCP overnight to abolish membrane potential and activate ISR. Immunoblotting revealed that these treatments did not alter GCN5L1 subcellular localization (Supplemental Figure 1G). However, GCN5L1 translocation was modulated by PA/OA treatment (Figure 1D), suggesting that the localization of GCN5L1 was regulated by fatty acid rather than mitochondrial ISR or dysfunction. The mechanisms of mitochondrial protein import have been well characterized (12). However, how mitochondrial proteins export to cytoplasm remains poorly understood. Given that the mitochondrial permeability transition pore (mPTP) is a Ca^2+^-dependent, cyclophilin D–facilitated (CypD-facilitated) channel in the inner mitochondrial membrane, its opening increases membrane permeability and permits the diffusion of molecules (e.g., mtDNA). We hypothesized that mitochondrial GCN5L1 could be released into the cytosol when the mPTP opens. The increased mPTP opening was confirmed by increased cytosolic mtDNA contents in PA/OA–treated hepatocytes. We found that cyclosporin A (CsA), a well-established inhibitor of mPTP opening (21), effectively reversed GCN5L1 mitochondrial localization in response to PA/OA treatment. Collectively, mPTP opening in response to PA/OA treatment mediates mitochondrial GCN5L1 release to cytoplasm, which would contribute to the increased cytosolic GCN5L1 in HFD feeding and PA/OA treatment (Supplemental Figure 1H).

In parallel, we generated an adeno-associated virus (AAV) with TBG promoter to overexpress GCN5L1-myc in mouse livers. When fed an NCD, AAV-GCN5L1-myc mice showed no significant difference in body weight or liver/body weight ratio, comparably to AAV-eGFP control mice. However, after 16 weeks of HFD, AAV-GCN5L1-myc mice exhibited higher body weight and liver/body weight ratio (Figure 1, F and G). Moreover, GCN5L1 overexpression significantly exacerbated HFD-induced hepatic TG content in comparison with controls (Figure 1H). Intriguingly, immunoblotting analysis revealed distinct localization patterns of GCN5L1-myc. In NCD-fed mice, GCN5L1-myc predominantly accumulated in mitochondria, whereas after HFD feeding, it showed minimal mitochondrial localization and was instead primarily present in the cytoplasm (Figure 1I). This striking correlation of cytoplasmic localization of GCN5L1 expression with hepatic lipid accumulation highlights an unrecognized role of GCN5L1 in MASLD (Figure 1J).

### GCN5L1 deletion protects mice from HFD-induced liver steatosis but not liver injury.

**Figure 2:**
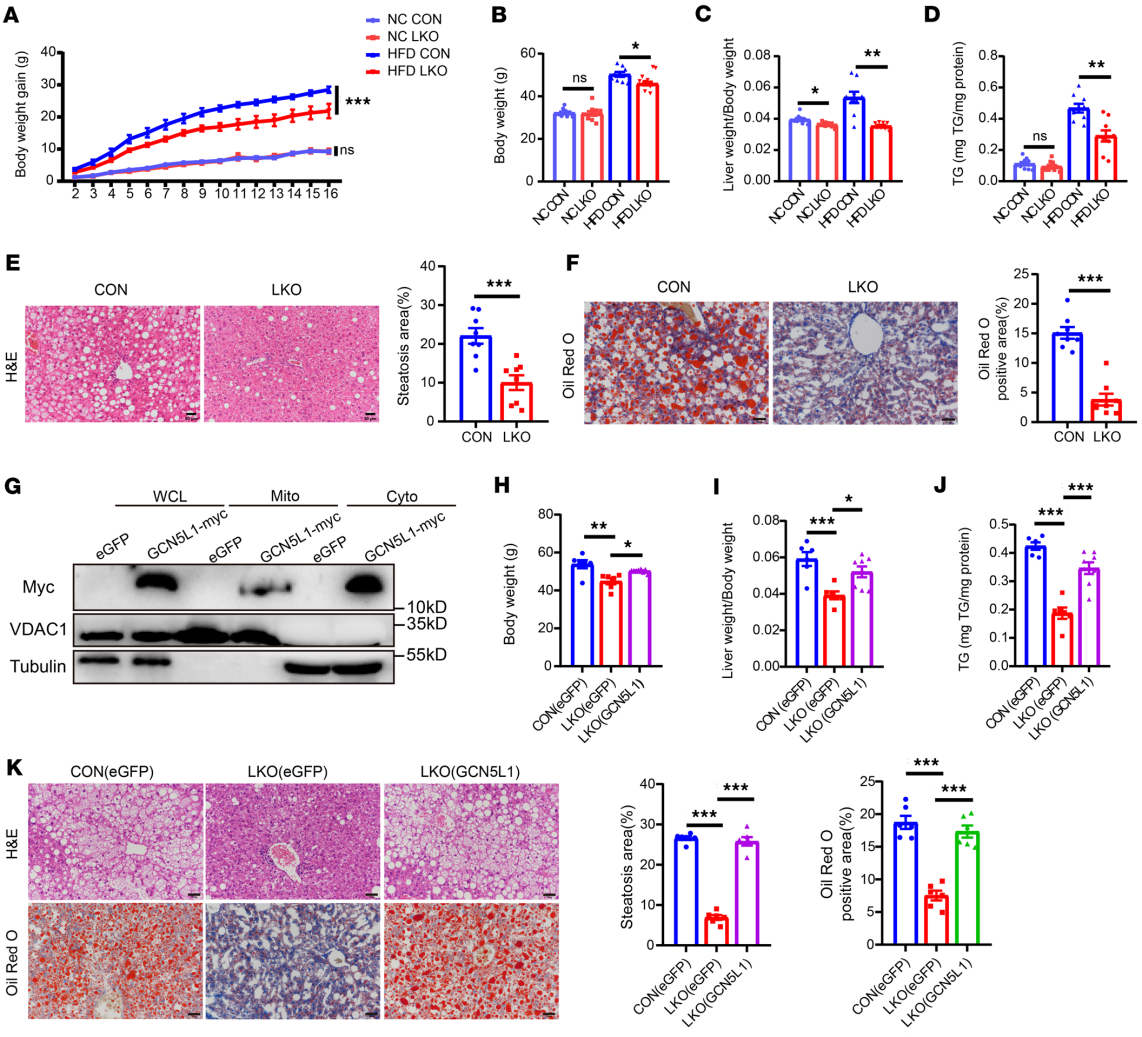
GCN5L1 deletion protects mice from HFD-induced liver steatosis but not liver injury. (**A**) Body weight gain of GCN5L1^fl/fl^ (CON) and GCN5L1^fl/fl^-Alb-Cre (LKO) mice on NC or HFD diets for 16 weeks. *n* = 10, NC; *n* = 9, CON with HFD; *n* = 10, LKO with HFD. (**B**) Body weights of CON or LKO mice on NC or HFD for 16 weeks. *n* = 10, NC; *n* = 9, HFD. (**C**) Liver/body weight ratio of CON or LKO mice on NC or HFD for 16 weeks. *n* = 10, NC; *n* = 9, HFD. (**D**) Liver TG contents of CON or LKO mice on NC or HFD for 16 weeks. *n* = 10, NC; *n* = 9, HFD. (**E**) Representative images of H&E staining of livers from CON or LKO mice on HFD for 16 weeks. Scale bars: 50 μm; *n* = 8 mice/group. (**F**) Representative images of Oil Red O staining of CON or LKO mice on HFD for 16 weeks. Scale bars: 50 μm; *n* = 8 mice/group. (**G**) Representative Western blot analysis of GCN5L1-myc in WCL, Mito, and Cyto fractions of liver samples from GCN5L1-LKO mice with AAV-eGFP or AAV-GCN5L1-myc expression. (**H**–**J**) GCN5L1 CON or LKO mice were injected with AAV-eGFP or AAV-GCN5L1-myc, followed by HFD feeding for 16 weeks. Body weight (**H**), liver/body weight ratio (**I**), and liver TG contents (**J**) were determined (*n* = 6, CON with AAV-eGFP; *n* = 6, LKO with AAV-eGFP; *n* = 8, LKO with AAV-GCN5L1-myc). (**K**) Representative images of H&E staining (top) or Oil Red O staining (bottom) of liver sections from CON-eGFP, LKO-eGFP, or LKO-GCN5L1-myc mice on HFD for 16 weeks. Scale bars: 50 μm; *n* = 6 mice/group. Data in **A**–**D** were derived from a single cohort of mice. Data in **H**–**J** pertain to an independent cohort of mice. Datasets in **A** were analyzed by 2-factor repeated-measures ANOVA with Bonferroni correction; in **B** and **H**–**K** were analyzed by 1-way ANOVA with Bonferroni correction; in **C** and **D** were analyzed by Welch’s ANOVA with Games-Howell post hoc tests; in **E** and **F** were analyzed by 2-tailed Student’s *t* test. ns, *P* > 0.05; *P < 0.05, **P < 0.01, ***P < 0.001.

Given that GCN5L1 translocated from mitochondria to cytoplasm during lipid overload (PA/OA incubation and HFD feeding), we hypothesized that cytoplasmic GCN5L1 had a distinct function in MASLD progression, rather than regulating mitochondrial FAO. To elucidate the function of cytoplasmic GCN5L1, we employed mice with hepatocyte-specific GCN5L1 knockout (GCN5L1 LKO) with HFD feeding to induce MASLD. GCN5L1-LKO mice exhibited body weight gain similar to that of GCN5L1-flox/flox (CON) mice upon NCD feeding. However, GCN5L1-LKO mice had significantly lower body weight gain under HFD (Figure 2A). GCN5L1-LKO mice showed reduced body weight and liver/body weight ratios after 16 weeks of HFD (Figure 2, B and C). Markers of hepatic steatosis, including hepatic TG content, H&E staining, and Oil Red O staining, all indicated that GCN5L1-LKO mice were protected from HFD-induced hepatic steatosis (Figure 2, D–F), consistent with previous reports (16). Leveraging this model, we hypothesized that the overexpressed GCN5L1 under HFD could retain GCN5L1 in the cytoplasm and restore liver TG contents. To test this, we injected 6-week-old GCN5L1-LKO mice with AAV-GCN5L1-myc via the tail vein. Two weeks after injection, the mice were subjected to HFD for 16 weeks. The cytoplasmic accumulation of GCN5L1-myc in HFD-fed LKO livers was confirmed by immunoblotting (Figure 2G), similar to the pattern observed in wild-type mice with GCN5L1 overexpression. Notably, GCN5L1-myc restoration rescued the body weight, liver/body weight ratios, and hepatic TG content in GCN5L1-LKO mice (Figure 2, H–J). H&E and Oil Red O staining further demonstrated that GCN5L1 overexpression reversed liver steatosis in HFD-fed GCN5L1-LKO mice (Figure 2K). These results suggest that HFD-induced cytoplasmic retention of GCN5L1 is critical for regulating hepatic steatosis.

Beyond liver steatosis, liver injury and fibrosis are common characteristics of MASLD. We assessed plasma transaminase activities, lipid parameters, and Sirius red staining of liver sections to evaluate liver injury and fibrosis. Plasma TG and total cholesterol (TC) levels were increased in GCN5L1-LKO mice compared with control mice under HFD (Supplemental Figure 2, A and B), suggesting increased lipoprotein release in GCN5L1-deleted livers. Moreover, plasma aspartate transaminase (AST) and alanine transaminase (ALT) levels, markers of liver injury, were higher in GCN5L1-LKO mice on either NCD or HFD, suggesting that GCN5L1 ablation induced liver injury (Supplemental Figure 2C). However, the collagen deposition indicated by Sirius red staining showed that GCN5L1 deletion did not affect liver fibrosis upon HFD feeding (Supplemental Figure 2D).

To further evaluate the function of hepatocyte GCN5L1 in MASH in vivo, we subjected GCN5L1-LKO and GCN5L1-flox/flox mice to a high-fat/high-cholesterol plus high-fructose (HFF) diet. GCN5L1-LKO mice showed lower body weight gain, body weight, and liver/body weight ratios than controls (Supplemental Figure 2, E–G). Hepatic deletion of GCN5L1 ameliorated liver steatosis, as evidenced by liver TG content and improved H&E staining (Supplemental Figure 2, H and I). Sirius red staining, fibrotic marker gene expression, and collagen immunoblotting indicated that GCN5L1-LKO also attenuated liver fibrosis in response to HFF feeding (Supplemental Figure 2, J–L).

Collectively, these findings demonstrate that cytoplasmic localization of GCN5L1 is essential for HFD-induced liver steatosis. The underlying regulatory mechanism appears to be independent of mitochondrial FAO, which is modulated by mitochondrial GCN5L1.

### GCN5L1 deletion inhibits de novo lipogenesis to reduce liver TG in response to HFD.

**Figure 3:**
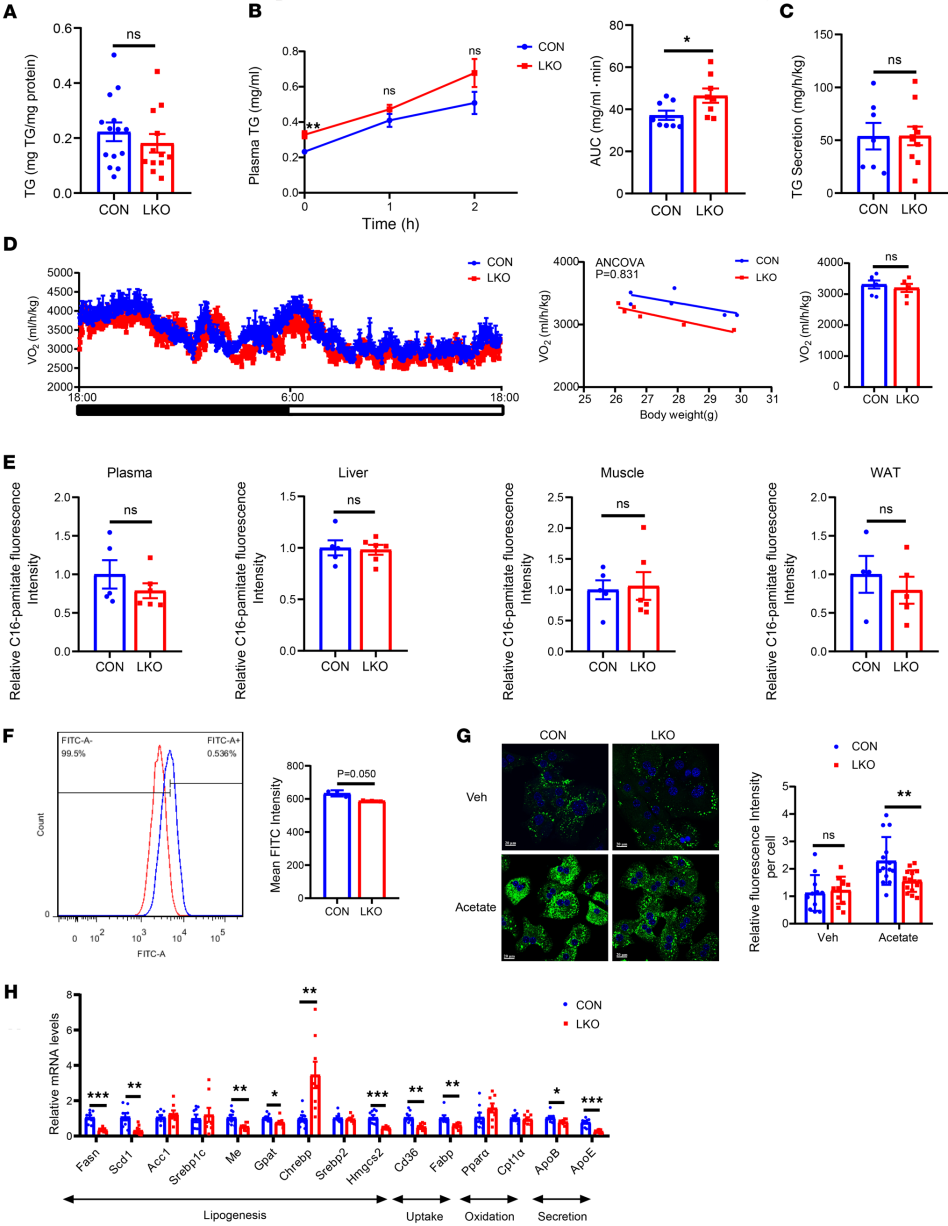
GCN5L1 deletion may inhibit de novo lipogenesis to reduce liver TG in response to HFD. (**A**) Feces TG of CON or LKO mice after 8 weeks of HFD (CON *n* = 14, LKO *n* = 12). (**B**) Plasma TG levels in HFD-fed CON or LKO mice (*n* = 8) orally receiving olive oil. (**C**) CON or LKO mice fed HFD for 8 weeks were fasted for 6 hours. Poloxamer 407 was administered via intraperitoneal injection (1 g/kg). Plasma TG levels were determined 2 hours after injection (CON *n* = 7, LKO *n* = 10). (**D**) Oxygen consumption was determined by metabolic cage experiments with CON or LKO mice on HFD. Oxygen consumption was normalized to body weight. ANCOVA analysis is shown on the right. (*n* = 6 mice per group.) (**E**) BODIPY-C16 fluorescence intensity in different tissues of CON or LKO mice (plasma, liver, muscle: CON *n* = 5, LKO *n* = 6; WAT: CON *n* = 4, LKO *n* = 5). WAT, white adipose tissue. (**F**) CON or LKO hepatocytes were analyzed by flow cytometry after incubation with 2 μM BODIPY-C16 for 10 minutes at 37°C (*n* = 3 mice per group). (**G**) Primary hepatocytes from CON and LKO mice were incubated with 40 mM acetate for 15 hours, followed by BODIPY staining and microscopy analysis; *n* > 10 cells from each group were analyzed. Scale bar: 20 μm. (**H**) Quantitative PCR detection of lipid metabolism–related genes in livers from CON or GCN5L1-LKO mice on HFD for 16 weeks (CON/LKO *n* = 8). Datasets in **A**, **C**, **E**, and **H** were analyzed by 2-tailed Student’s *t* test. Datasets in **B** were analyzed by 2-factor repeated-measures ANOVA with Bonferroni correction. Datasets in **F** were analyzed by non-parametric statistical tests. Datasets in **G** were analyzed by 2-way ANOVA with Bonferroni correction. ns, *P* > 0.05; *P < 0.05, **P < 0.01, ***P < 0.001.

Several processes are critical for liver steatosis, including increased de novo lipogenesis in hepatocytes and fatty acid uptake from circulation, decreased lipid catabolism through FAO, and very low-density lipoprotein release. To elucidate the pathway(s) mediating GCN5L1 deletion, which is attenuated in liver steatosis, we first measured fecal TG levels in GCN5L1-LKO and control mice, ruling out intestinal absorption as a confounding factor. The fecal levels were detected with no differences in TG levels (Figure 3A). We observed reduced hepatic TG content but increased plasma TG levels in GCN5L1-LKO mice with HFD, suggesting the imbalance of hepatic TG secretion and uptake by peripheral tissues, including muscle, adipose, and liver itself. Given cytoplasmic GCN5L1 accumulation starting at 4 weeks of HFD, we performed subsequent experiments with mice fed HFD for 4–8 weeks with comparable body weights. A lipid load assay showed that GCN5L1-LKO mice had higher baseline plasma TG, and their TG clearance rates were slightly worse than controls (Figure 3B).

Next, we examined TG secretion rate by injecting the mice with poloxamer 407, a non-ionic detergent that can block TG uptake in mice. Injection of poloxamer 407 confirmed unaltered hepatic TG secretion (Figure 3C). We then determined whether GCN5L1 deletion enhances FAO. The metabolic cage was introduced to detect oxygen consumption in GCN5L1-LKO and control mice upon HFD; the comparable ANCOVA of oxygen consumption rate between these 2 groups of mice excluded enhanced FAO as the primary mechanism of reduction of hepatic TG (Figure 3D). Our and others’ previous works demonstrate that mitochondrial GCN5L1 regulates FAO in HepG2 cells and primary hepatocytes in a manner partially dependent on the deacetylation regulation of mitochondrial proteins, e.g., CPT1, LCAD, and HADHA (16, 22). Therefore, during the development of MASLD, the decrease of mitochondrial localized GCN5L1 is thought to enhance FAO, which could not account for TG accumulation in MASLD, suggesting that cytoplasmic accumulated GCN5L1 plays a crucial role in mediating the impact of GCN5L1 on liver steatosis.

We aimed to elucidate whether the alleviated liver steatosis in GCN5L1-LKO mice resulted from decreased fatty acid uptake or de novo lipogenesis. BODIPY FL-C16, a fluorescent analog of long-chain fatty acids (23, 24), was used to monitor fatty acid uptake both in vivo and in vitro. GCN5L1-LKO and control mice were intraperitoneally injected with BODIPY FL-C16, and 2 hours later, plasma, liver, and other tissues were collected for fluorescence detection to assess fatty acid uptake. Fluorescence intensities in the plasma, liver, and other tissues were comparable between GCN5L1-LKO and CON mice (Figure 3E). In the in vitro assay, primary hepatocytes from GCN5L1-LKO and CON mice were incubated with BODIPY FL-C16, followed by flow cytometry analysis. The results showed a negligible reduction in hepatocyte uptake (Figure 3F). These data suggested that decreased FFA uptake did not account for reduced hepatic TG in GCN5L1-LKO mice.

Acetyl-CoA is the substrate for de novo lipogenesis, while acetate supplement increases acetyl-CoA levels in cytoplasm and enhances genome acetylation to activate lipogenesis (25, 26). Hepatocytes were incubated with 40 mM acetate for 15 hours, and lipid droplets were visualized by BODIPY staining. Acetate incubation significantly enhanced lipid accumulation in CON hepatocytes. In contrast, GCN5L1 deletion inhibited lipid droplet formation (Figure 3G), suggesting that GCN5L1 deletion inhibits de novo lipogenesis. The expression of key enzymes involved in de novo lipogenesis, fatty acid uptake, and TG secretion, such as Fasn, Scd1, Gpat, Cd36, and ApoE, was significantly downregulated in GCN5L1-LKO mice, whereas the expression of FAO showed no changes (Figure 3H). Collectively, these results demonstrate that GCN5L1 deletion suppresses de novo lipogenesis in the livers of HFD-fed mice.

### GCN5L1 deletion reduces PPARγ and its downstream gene expression.

**Figure 4:**
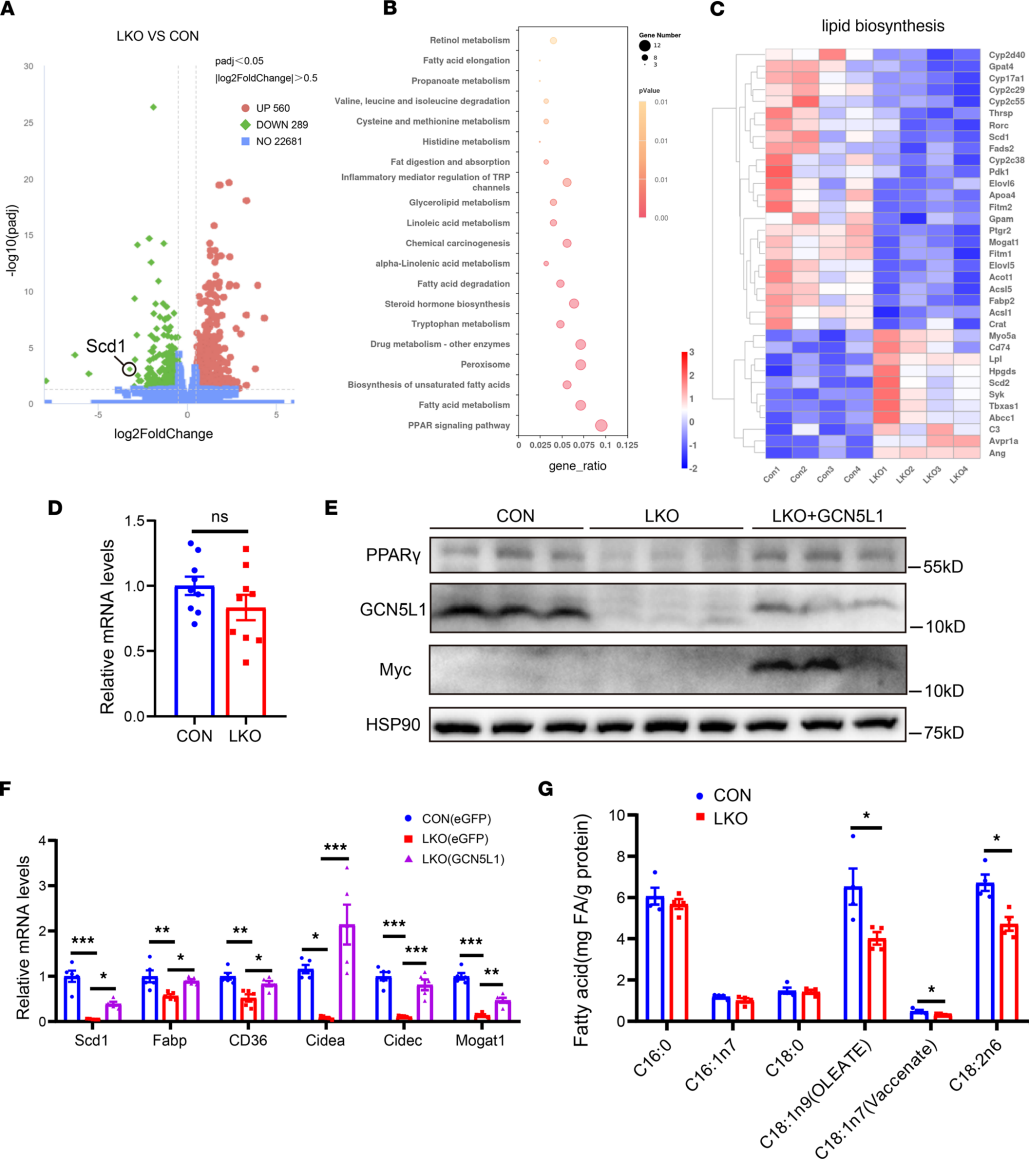
GCN5L1 deletion reduces PPARγ and its downstream gene expression. (**A**) RNA sequencing and analysis of differential gene expressions in livers from CON or GCN5L1-LKO mice on HFD for 16 weeks. (**B**) KEGG analysis of all downregulated genes from RNA sequencing. (**C**) Heatmap analysis of differential genes of lipid synthesis from RNA sequencing. (**D**) PPARγ RNA levels in CON or GCN5L1-LKO mice on HFD for 16 weeks (*n* = 9 mice per group). (**E**) PPARγ protein levels in livers from CON, GCN5L1-LKO, or LKO-AAV-GCN5L1 mice with HFD. (**F**) Quantitative PCR detection of target genes downstream of PPARγ in CON, GCN5L1-LKO, or LKO-AAV-GCN5L1 mice on HFD for 16 weeks (*n* = 5 mice per group). (**G**) Analysis of lipid profile in livers from CON and GCN5L1-LKO mice (*n* = 4 mice per group). For comparison between 2 groups in which *n* was 4, datasets were analyzed by 1-way ANOVA with Dunnett’s post hoc test. Datasets in **D** were analyzed by 2-tailed Student’s *t* test. Datasets in **F** were analyzed by 1-way ANOVA with Bonferroni correction. Datasets in **G** were analyzed by non-parametric statistical tests. ns, *P* > 0.05; *P < 0.05, **P < 0.01, ***P < 0.001.

To investigate the mechanism(s) by which GCN5L1 regulates hepatic steatosis, particularly the lipogenic pathway, we conducted a transcriptomic analysis of liver tissues from HFD-fed GCN5L1-LKO mice and control mice. The analysis revealed that 560 genes were upregulated and 289 genes were downregulated in GCN5L1-LKO livers. Kyoto Encyclopedia of Genes and Genomes (KEGG) analysis of downregulated genes showed a significant enrichment in PPAR signaling pathway, fatty acid metabolism, and biogenesis of unsaturated fatty acids (Figure 4, A and B). Further examination of lipid biosynthesis–related genes demonstrated that most of these genes were downregulated in GCN5L1-LKO mice, including key enzymes such as Scd1, Elov6, Acsl5, and Gpat (Figure 4C). Meanwhile, the upregulated genes were enriched in the phagosome and lysosome pathways (Supplemental Figure 3A). Based on these findings, we hypothesized that deletion of GCN5L1 inhibits the PPARγ pathway, thereby alleviating liver steatosis. Notably, the most predominant transcriptional factors involved in overnutrition-induced liver steatosis are Srebp1, ChREBP, FXR, etc. (27, 28). PPARγ is a key transcriptional factor to induce lipogenesis in adipose tissues (29), although its functions in the liver have been a subject of controversy (30–35). To explore whether and how the PPARγ pathway regulates lipid metabolism in GCN5L1-LKO livers, we first assessed the transcriptional activity of PPARγ in GCN5L1-deleted and control cells. The PPARγ activity luciferase construct was transfected into AML12 cells with GCN5L1 deletion by sgRNA or control. The transcriptional activity of PPARγ was found to be decreased in GCN5L1-knockout (KO) cells, and this difference was further amplified by incubation with PA/OA (Supplemental Figure 3B). Moreover, luciferase activity exhibited a proportional rise with GCN5L1 expression during PA/OA incubation (Supplemental Figure 3C). In addition, rosiglitazone, the PPARγ agonist, promoted PPARγ activity in both control and GCN5L1-KO cells (Supplemental Figure 3D); however, PPARγ activity in KO cells remained lower even after rosiglitazone treatment, suggesting that GCN5L1 deletion might affect the protein levels of PPARγ. Therefore, we evaluated the transcriptional and protein levels of PPARγ in GCN5L1-LKO livers. RNA sequencing data identified comparable RNA levels of PPARγ in GCN5L1-LKO and CON mice, which was confirmed by qPCR (Figure 4D). We then evaluated the protein levels of PPARγ in livers of GCN5L1-LKO and control mice fed an HFD, as well as in primary hepatocytes treated with PA/OA. The protein levels of PPARγ were significantly decreased in GCN5L1-LKO livers, and this decrease was reversed by AAV-GCN5L1-myc expression in livers (Figure 4E). GCN5L1 deletion also led to a reduction in PPARγ protein levels in response to PA/OA treatment (Supplemental Figure 3E). In parallel, the expression of PPARγ downstream genes, including Scd1, Fabp4, Cd36, Cidea, Cidec, and Mogat1 (33, 36), was confirmed by RT-PCR, which were downregulated in GCN5L1-LKO livers and restored by GCN5L1 overexpression (Figure 4F). These data indicate that the decreased protein level of PPARγ mediates the reduction of hepatic TG in GCN5L1-LKO mice upon HFD feeding.

Furthermore, the rate-limiting enzymes that regulate the synthesis of monounsaturated fatty acids — stearoyl-coenzyme A desaturases, especially SCD1, a classical target gene downstream of PPARγ — were dramatically decreased in GCN5L1-LKO livers (Figure 4A). Previous reports have associated SCD1 with the alleviation of liver steatosis but worse liver injury upon HFD (37–39). We hypothesized that the insufficient transcriptional levels of SCD1 inhibited lipid accumulation in GCN5L1-LKO livers with HFD feeding. To test this, we first examined the protein levels of SCD1, which was dramatically downregulated in GCN5L1-LKO livers (Supplemental Figure 4A). We then analyzed the lipid profile in livers from GCN5L1-LKO and CON mice. Liver extracts were subjected to LC-MS to quantify all fatty acids. The total hepatic fatty acid levels were comparable, and the levels of saturated fatty acids, such as palmitic acid, remained unchanged. However, the levels of unsaturated fatty acids, particularly C18:1 and C18:2, were significantly reduced in GCN5L1-LKO livers, indicating that the synthesis of unsaturated fatty acids was impaired in the absence of GCN5L1 (Figure 4G). We then investigated whether the decrease of Scd1 contributed to the attenuation of liver steatosis in GCN5L1-LKO mice on an HFD. We generated AAV-Scd1-FLAG and administered AAV-Scd1-FLAG or eGFP control to GCN5L1-LKO mice via tail vein injection. The mice were subsequently fed an HFD for 16 weeks, and the liver TG levels and plasma AST and ALT levels were measured. Scd1 expression could not restore the liver TG levels in GCN5L1-LKO mice; however, the deficiency of unsaturated fatty acids might be responsible for the liver injury observed in GCN5L1-LKO mice (Supplemental Figure 4, B–D).

### GCN5L1 deletion modulates PPARγ stability via ubiquitylation-mediated proteasome degradation.

**Figure 5:**
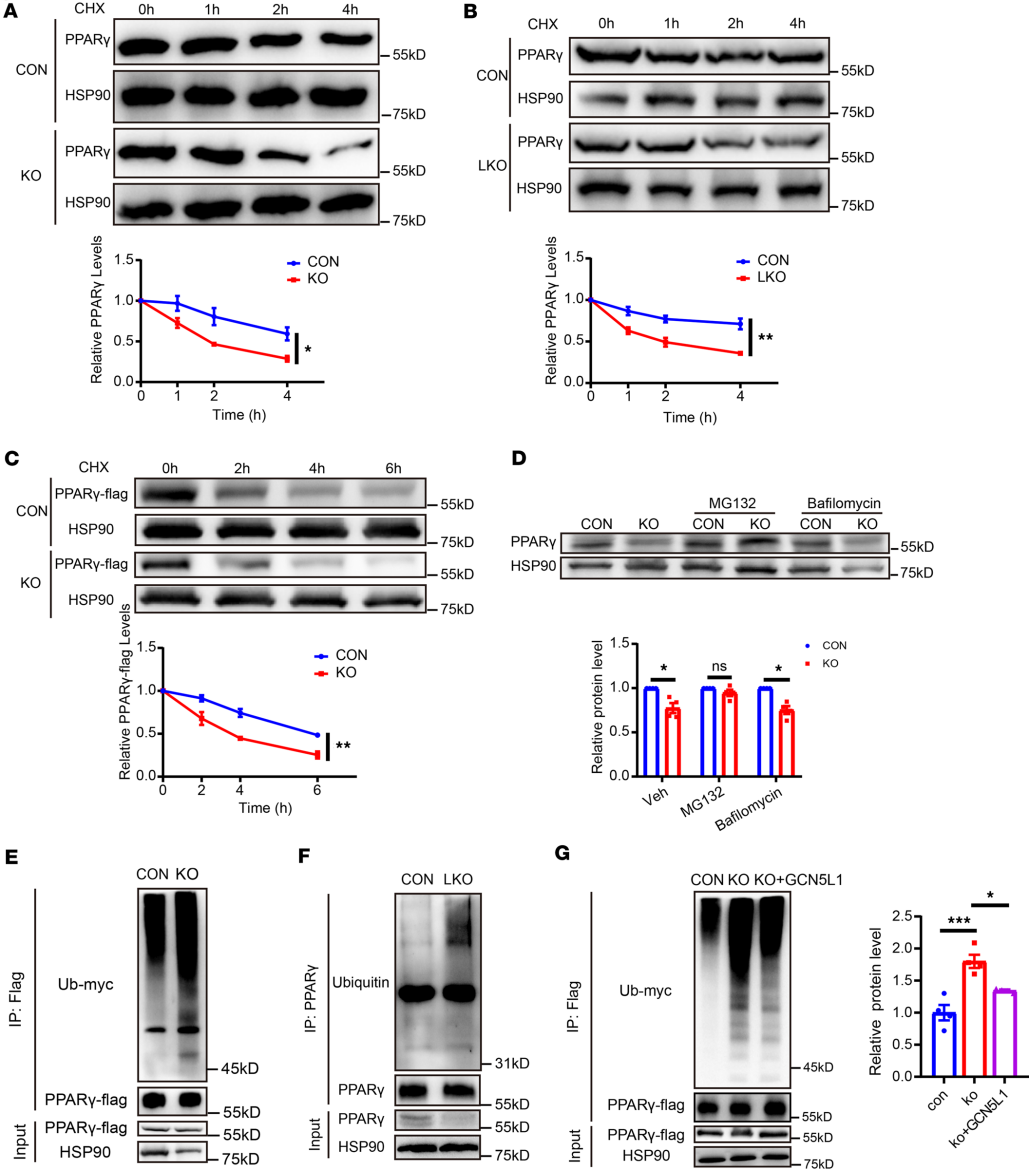
GCN5L1 deletion modulates PPARγ stability via ubiquitylation-mediated proteasome degradation. (**A**) GCN5L1 deletion (KO) or control (CON) C3H10T1/2 cells were stimulated with 50 μg/mL cycloheximide (CHX) for 0, 1, 2, and 4 hours, followed by Western blot analysis of PPARγ. Quantitation of 4 independent experiments is shown. (**B**) Primary hepatocytes from CON or GCN5L1-LKO mice were incubated with 0.4 mM PA/0.8 mM OA overnight, followed by stimulation with 50 μg/mL CHX for 0, 1, 2, and 4 hours. Western blot was used to detect PPARγ. Quantitation of 4 independent experiments is shown. (**C**) HepG2 cells with or without GCN5L1 deletion were transfected with PPARγ-FLAG. PPARγ-FLAG levels were assessed via Western blot analysis after CHX stimulation. Quantitation of 4 independent experiments is shown. (**D**) C3H10T1/2 cells with or without GCN5L1 deletion were incubated with MG132 (10 μM) or bafilomycin (20 nM). Western blot was used to detect PPARγ. Quantitation of 4 independent experiments is shown. (**E**) The PPARγ-FLAG and ubiquitin-myc (Ub-myc) plasmids were cotransfected into HepG2 CON or KO cells. PPARγ-FLAG was immunoprecipitated by anti-FLAG antibody. Ubiquitination levels were analyzed by immunoblotting with anti-myc antibody. (**F**) PPARγ antibody was used for immunoprecipitation of endogenous PPARγ. Ubiquitination levels were analyzed by immunoblotting in livers from CON or GCN5L1-LKO mice with 16-week HFD. (**G**) The PPARγ-FLAG and Ub-myc plasmids were cotransfected into HepG2 CON, GCN5L1-KO, or KO-lenti-GCN5L1 cells. PPARγ-FLAG was immunoprecipitated by anti-FLAG antibody. Ubiquitination levels were analyzed by immunoblotting with anti-myc antibody. Quantitation of 4 independent experiments is shown. Datasets in **A**–**C** were analyzed by 2-factor repeated-measures ANOVA with Bonferroni correction. Datasets in **D** were analyzed by 2-way ANOVA with Bonferroni correction. Datasets in **G** were analyzed by 1-way ANOVA with Bonferroni correction. ns, *P* > 0.05; *P < 0.05, **P < 0.01, ***P < 0.001.

Next, we investigated the mechanisms underlying the reduction of PPARγ protein levels in GCN5L1-LKO livers. Given that PPARγ protein levels but not RNA levels were decreased in GCN5L1-LKO livers, we hypothesized that GCN5L1 deletion impacts PPARγ protein stability. To test this, we used differentiated C3H10T1/2 cells to analyze PPARγ protein turnover. GCN5L1-KO and control cells were incubated with the protein synthesis inhibitor cycloheximide. Immunoblotting confirmed a decrease in PPARγ protein levels in C3H10T1/2 cells with or without GCN5L1 deletion (Supplemental Figure 5A). Notably, the rate of PPARγ degradation was significantly higher in GCN5L1-KO C3H10T1/2 cells and in primary hepatocytes from GCN5L1-LKO livers with PA/OA treatment (Figure 5, A and B). Exogenous PPARγ degradation was also observed in HepG2 cells with GCN5L1 deletion (Figure 5C). Collectively, these findings indicate that PPARγ protein stability is substantially reduced in GCN5L1-deleted cells.

The ubiquitin-proteasome system and the autophagy-lysosome system are the two major protein degradation pathways in cells (40). To determine the degradation pathway(s) involved following GCN5L1 depletion, we treated differentiated C3H10T1/2 cells with bafilomycin A1 (an endosome/lysosome inhibitor) or MG132 (a proteasome inhibitor) and then evaluated PPARγ protein levels. MG132, but not bafilomycin A1, prevented PPARγ degradation in GCN5L1-deleted cells (Figure 5D), suggesting that proteasomal degradation contributes to decreased PPARγ protein levels in GCN5L1-KO cells. As ubiquitination is the predominant factor in proteasome-mediated degradation (40), we assessed the extent of PPARγ ubiquitylation in the presence of MG132. A notable increase in the ubiquitination levels of PPARγ was observed in GCN5L1-KO HepG2 cells expressing PPARγ-FLAG (Figure 5E). Similarly, endogenous PPARγ was immunoprecipitated from HFD-fed mouse livers from GCN5L1-LKO and CON mice; PPARγ ubiquitination levels were markedly elevated in GCN5L1-LKO livers (Figure 5F). A similar result was obtained in differentiated C3H10T1/2 cells (Supplemental Figure 5B). Moreover, restoration of GCN5L1 expression in PA/OA–treated HepG2 cells reversed the increase in PPARγ ubiquitination levels (Figure 5G). Taken together, these results demonstrate that GCN5L1 deletion promotes PPARγ ubiquitination and subsequent degradation via the proteasome system.

### GCN5L1 modulates PPARγ-K289 acetylation to prevent ubiquitination and degradation.

**Figure 6:**
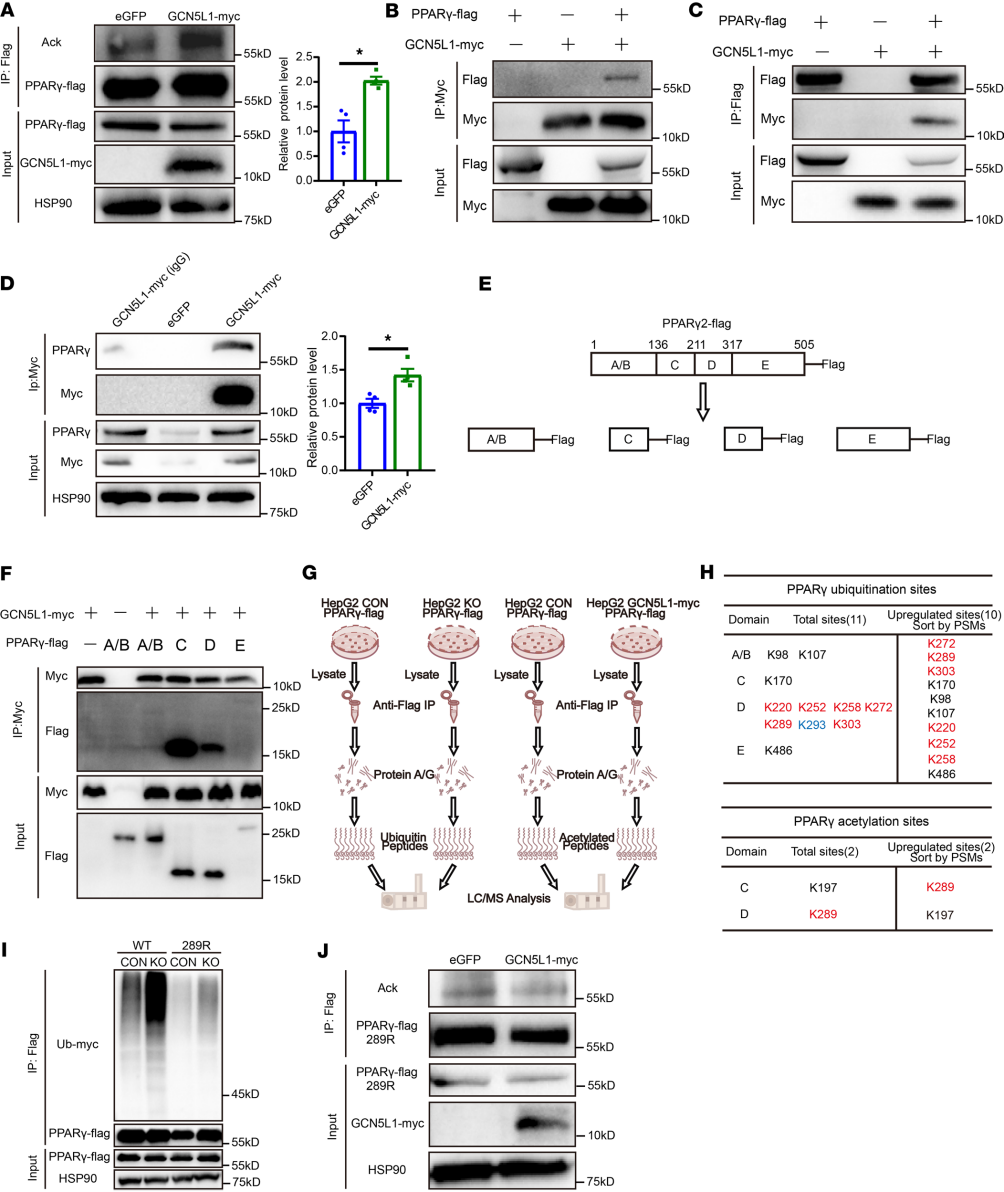
GCN5L1 modulates PPARγ-K289 acetylation to prevent ubiquitination and degradation. (**A**) HepG2 control or GCN5L1-myc–overexpressing cells were transfected with PPARγ-FLAG. Immunoprecipitation was performed using a FLAG antibody to detect the acetylation level of PPARγ-FLAG. Quantitation of 4 independent experiments is shown. (**B**) Coimmunoprecipitation (co-IP) was performed to validate the interaction between PPARγ-FLAG and GCN5L1-myc. Immunoprecipitation was conducted using an anti-myc antibody. (**C**) Co-IP assays were performed to validate the interaction between PPARγ-FLAG and GCN5L1-myc. Immunoprecipitation was conducted using an anti-FLAG antibody. (**D**) Co-IP was performed to validate the interaction between PPARγ and GCN5L1-myc. GCN5L1-myc was precipitated from AAV-GCN5L1-myc or AAV-eGFP mice. PPARγ was assessed by immunoblotting. Quantitation of input PPARγ levels in livers from AAV-GCN5L1-myc or AAV-eGFP mice is shown (right) (data from 4 independent experiments). (**E**) Illustration indicates domains of PPARγ. (**F**) Co-IP validation of the interaction of PPARγ-FLAG domains with GCN5L1-myc. (**G**) Illustration of the procedures to identify ubiquitination or acetylation residues of PPARγ by immunoprecipitation coupled with mass spectrometry. (**H**) PPARγ ubiquitination or acetylation residues were detected in HepG2 cells. Red marks the increased modification in GCN5L1-KO cells for ubiquitinated lysine residues or in GCN5L1-myc cells for acetylated lysine residues in D domain; blue-labeled K293 exhibited no significant variance between the control and GCN5L1 KO. PSM, peptide spectrum match. (**I**) PPARγ-wild-type or -K289R plasmids, along with the ubiquitin-myc (Ub-myc) plasmid, were cotransfected into HepG2 CON or GCN5L1-KO cells. PPARγ-FLAG or PPARγ-K289R-FLAG was immunoprecipitated to assess the ubiquitination levels by immunoblotting. (**J**) HepG2 CON or GCN5L1-myc–overexpressing cells were transfected with PPARγ-K289R-FLAG. PPARγ-K289R-FLAG was immunoprecipitated to assess the acetylation levels by immunoblotting. Datasets in **A** and **D** were analyzed by non-parametric statistical tests. *P < 0.05.

Ubiquitination and acetylation are posttranslational modifications occurring on lysine residues. Given that GCN5L1 functions as a regulator of protein acetylation in mitochondria, we hypothesized that cytoplasmic GCN5L1 might modulate PPARγ acetylation, thereby competitively inhibiting ubiquitination on the same lysine residues. To test this hypothesis, we first investigated whether GCN5L1 regulates PPARγ acetylation. PPARγ-FLAG was transfected into HepG2 cells with or without GCN5L1 overexpression. Considering that PA/OA treatment mimics HFD feeding and promotes GCN5L1 cytoplasmic localization, the cells were treated with PA/OA for 16 hours. PPARγ was then immunoprecipitated using anti-FLAG resin, and its acetyl-lysine levels were evaluated by immunoblotting. GCN5L1 overexpression significantly enhanced PPARγ acetylation (Figure 6A). To further confirm this, we adapted an in vitro assay using purified proteins and acetyl-CoA in pH 7.4 buffer to mimic the cytosolic environment. FLAG-tagged PPARγ was incubated with BL21-derived GCN5L1 in the presence or absence of acetyl-CoA. Immunoblotting results indicated that significant levels of PPARγ acetylation were detectable under incubation with 1.5 mM acetyl-CoA. Coincubation with GCN5L1 further increased PPARγ acetylation (Supplemental Figure 6A). Next, we examined the interaction between GCN5L1 and PPARγ. Coimmunoprecipitation (co-IP) assays in HepG2 cells overexpressing both proteins confirmed their interaction (Figure 6, B and C). Additionally, GCN5L1 was immunoprecipitated by a myc antibody from livers of mice expressing AAV-GCN5L1-myc and fed an HFD for 4 weeks. Immunoblotting analysis further validated the interaction between GCN5L1 and PPARγ in these liver samples (Figure 6D). Interestingly, PPARγ protein levels were increased in livers of mice expressing GCN5L1-myc (Figure 6D). Collectively, these findings indicate that GCN5L1 interacts with PPARγ and modulates its acetylation.

To determine whether GCN5L1 competitively regulates PPARγ acetylation to block its ubiquitination, we used proteomic analysis to study PPARγ modifications. As reported in the literature, PPARγ contains 4 domains: the A/B domain encompasses a ligand-independent transcriptional activation function; the C domain contains a DNA-binding domain capable of targeting specific DNA sequences; the D domain, constituting the hinge region, is critical for cofactor docking. The E/F region is a ligand-binding structural domain that can be activated by natural or synthetic ligands (41). We generated truncates of PPARγ containing the A/B, C, D, and E domains (Figure 6E). First, we identified the domains of PPARγ that interacted with GCN5L1. By expressing PPARγ truncates along with GCN5L1 in HepG2 cells and performing co-IP assays, we found that GCN5L1 primarily interacts with the C and D domains of PPARγ (Figure 6F). Then we used 2 systems to identify PPARγ modifications regulated by GCN5L1. In the first system, HepG2 cells were generated with stable expression of PPARγ-FLAG in GCN5L1-KO or CON backgrounds to analyze ubiquitination. In the second system, cells stably expressing PPARγ-FLAG and GCN5L1-myc were treated with PA/OA to mimic HFD feeding, enabling the analysis of acetylation. PPARγ-FLAG was accumulated by immunoprecipitation, and mass spectrometry was used to identify the modifications regulated by GCN5L1 (Figure 6G). Mass spectrometry analysis identified 11 lysine residues undergoing ubiquitination. Among them, 9 residues were uniquely ubiquitinated in GCN5L1-deleted cells, and 10 residues showed increased quantification in GCN5L1-KO cells compared with control cells. Lysine at positions K272, K289, and K303 exhibited the top 3 ubiquitination levels relative to other residues (Figure 6H). Additionally, 2 acetylated lysine residues were detected, and acetylation of PPARγ at K289 was increased in GCN5L1-overexpressed cells (Figure 6H). Interestingly, 7 of 11 ubiquitinated residues (including the top 3) were localized in the D domain, as was the only acetylation residue regulated by GCN5L1. This shared locus in both ubiquitination and acetylation analyses suggests its critical role as a key target of GCN5L1 in PPARγ regulation.

To further validate our findings, we constructed a plasmid with a mutation at the PPARγ 289 locus, altering the lysine to arginine to mimic deacetylation (Supplemental Figure 6B). PPARγ wild-type plasmid or PPARγ-K289R mutant construct was transfected into GCN5L1-KO or CON HepG2 cells, followed by overnight treatment with PA/OA and MG132. The results showed that the ubiquitination levels of wild-type PPARγ were significantly elevated in GCN5L1-deleted cells (Figure 6I). In contrast, the ubiquitination of PPARγ-K289R mutation was significantly downregulated in comparison with wild-type PPARγ in both CON and GCN5L1-KO cells, indicating that K289 is essential for PPARγ ubiquitination and degradation (Figure 6I). Moreover, when PPARγ-K289R was expressed in HepG2 cells with or without GCN5L1-myc overexpression, the acetylation levels of PPARγ-K289R were comparable between the two groups, further confirming that GCN5L1 regulates PPARγ acetylation at K289 (Figure 6J). In conclusion, GCN5L1 promotes acetylation of PPARγ at K289, which competitively blocks ubiquitination at this site, thereby reducing PPARγ degradation.

### PPARγ-K289 mutation increases PPARγ stability and liver steatosis in HFD mice.

**Figure 7:**
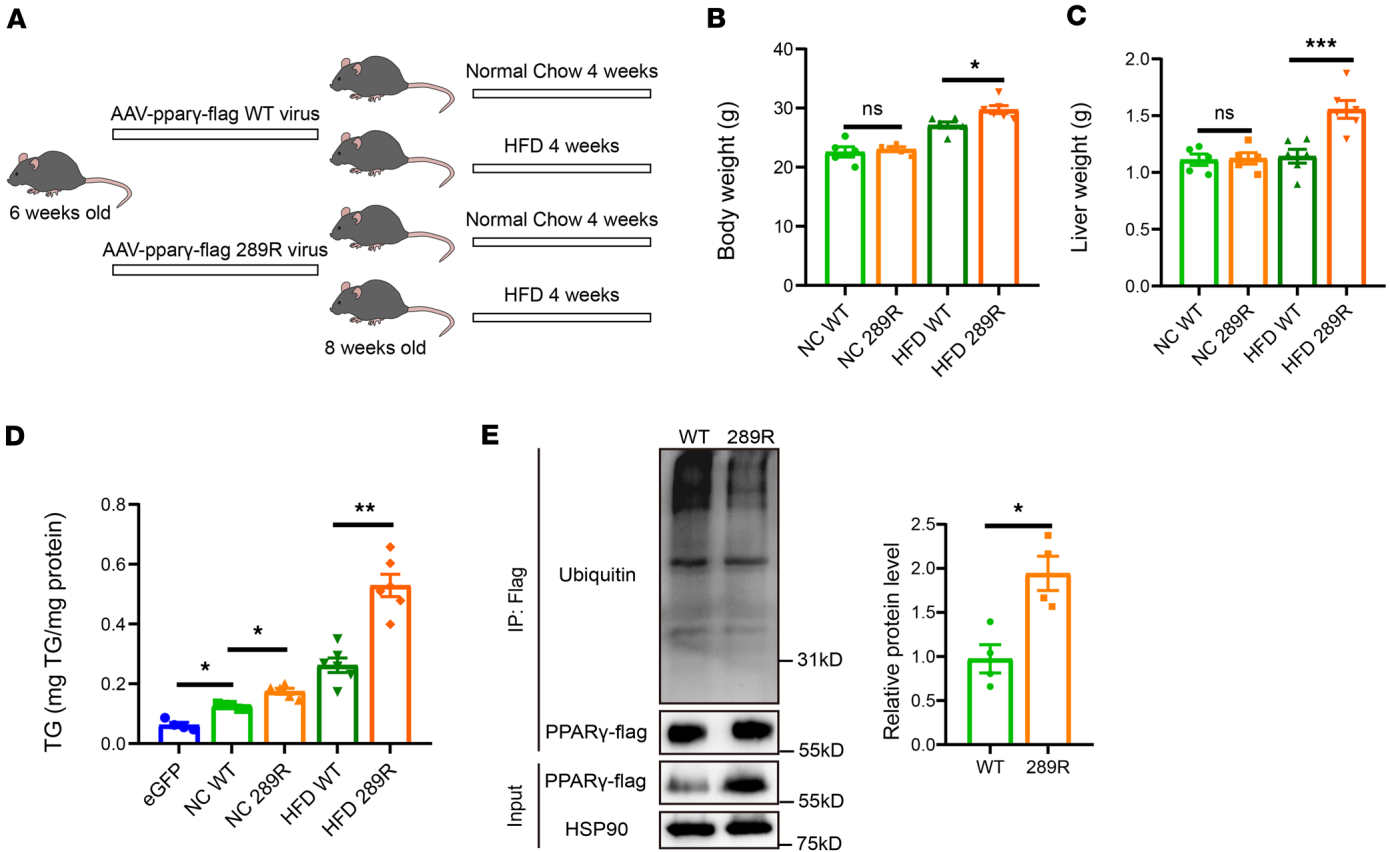
PPARγ-K289 mutation increases PPARγ stability and liver steatosis in HFD mice. (**A**) C57BL/6 mice were given AAV-PPARγ-FLAG or AAV-PPARγ-K289R-FLAG via tail vein injection. The mice were subjected to either NC or HFD for 4 weeks and analyzed for liver TG content and PPARγ ubiquitination (*n* = 5 mice per group for NC; *n* = 6 mice per group for HFD). (**B**) Body weights of PPARγ-FLAG or PPARγ-K289R-FLAG mice on NC or HFD for 4 weeks (*n* = 5 mice per group for NC; *n* = 6 mice per group for HFD). (**C**) Liver weights of PPARγ-FLAG or PPARγ-K289R-FLAG mice on NC or HFD for 4 weeks (*n* = 5 mice per group for NC; *n* = 6 mice per group for HFD). (**D**) Liver TG content of eGFP, PPARγ-FLAG, or PPARγ-K289R-FLAG mice on NC or HFD as indicated (*n* = 4 mice per group for AAV-eGFP mice on NC; *n* = 5 mice per group for AAV-PPARγ mice on NC; *n* = 6 mice per group for HFD). (**E**) PPARγ-FLAG and PPARγ-K289R-FLAG were precipitated from mouse livers, and ubiquitination levels were detected by immunoblotting. Quantitation of 4 independent experiments is shown. Datasets in **B** and **C** were analyzed by 2-way ANOVA with Bonferroni correction. Datasets in **D** were analyzed by Welch’s ANOVA with Games-Howell post hoc tests. Datasets in **E** were analyzed by non-parametric statistical tests. ns, *P* > 0.05; *P < 0.05, **P < 0.01, ***P < 0.001.

To investigate the physiological function of PPARγ-K289, we generated AAV-TBG-PPARγ and AAV-TBG-PPARγ-K289R to express wild-type PPARγ and its mutant in mouse livers. Given our hypothesis that cytoplasmic GCN5L1 serves as a major regulator of PPARγ-K289 acetylation and considering that 4 weeks of HFD is required to ensure cytoplasmic GCN5L1 accumulation, we conducted the following experiments. Wild-type mice at 6 weeks were injected with AAV-TBG-PPARγ or AAV-TBG-PPARγ-K289R via tail vein and subjected to NCD or HFD for 4 weeks (Figure 7A). Two weeks after AAV injection, immunoblotting analysis confirmed the protein expression of both wild-type PPARγ and PPARγ-K289R in mice on the NCD (Supplemental Figure 7A). PPARγ-K289R mice exhibited comparable body weight and liver weight when the mice were on NCD. However, under HFD feeding, both groups exhibited increased body weight, with PPARγ-K289R mice showing significantly higher body weight and liver weight than wild-type mice (Figure 7, B and C). Notably, PPARγ wild-type expression enhanced hepatic TG content compared with eGFP control even when the mice were on NCD, suggesting that PPARγ promotes lipogenesis in livers (Figure 7D). HFD further exacerbated liver TG accumulation, which was significantly more pronounced in PPARγ-K289R–expressing mice than in wild-type mice (Figure 7D). This phenotype correlated with higher protein levels of PPARγ-K289R, which was confirmed to possess reduced ubiquitination (Figure 7E). Plasma TG levels remained unaffected by PPARγ-K289 mutation regardless of diet condition (Supplemental Figure 7B). However, plasma cholesterol levels and AST and ALT levels were significantly enhanced in PPARγ-K289R mice compared with PPARγ-wild-type mice on HFD (Supplemental Figure 7, C and D). These phenotypes were similar to those observations in GCN5L1-LKO mice, suggesting that deacetylation of PPARγ-K289 is a key mediator of GCN5L1-regulated hepatic lipid metabolic disorders. Collectively, these results demonstrate that the acetylation of PPARγ-K289 is critical for its stability in vivo and plays a pivotal role in promoting liver TG accumulation, particularly under HFD conditions.

## Discussion

In the current study, we aimed to explore whether the mitochondrial protein GCN5L1 mediates cytoplasmic events and, if so, how it regulates hepatic lipid homeostasis during the progression of metabolic dysfunction–associated steatotic liver disease (MASLD). We first assessed the transcriptional and protein levels of GCN5L1 in MASLD patients and mouse models. Our findings revealed that GCN5L1 expression was elevated in MASLD liver tissues. Interestingly, the redistribution of GCN5L1, characterized by a significant increase in its cytoplasmic localization, was strongly correlated with liver steatosis. This redistribution supports the idea that the role of GCN5L1 in hepatic lipid metabolism extends beyond its involvement in mitochondria-associated FAO, implicating it in the fatty acid synthesis process. Concurrently with the reduction of mitochondrially localized GCN5L1 during MASLD, the mitochondrial protein acetylation was found to be decreased while cytosolic protein acetylation was moderately enhanced. These changes further imply that GCN5L1 functions as a regulator of protein acetylation in cytoplasm. Indeed, GCN5L1 has previously been detected in the cytoplasm, as a component of BLOC1 and BORC complexes, where it participates in lysosome trafficking and lysosome-associated lipid oxidation (13, 17, 18); its role in cytoplasmic acetylation was previously thought to be limited to tubulin acetylation, with other potential substrates remaining largely uncharacterized. Here, we demonstrated that cytoplasmic GCN5L1 modulates de novo fatty acid synthesis via directly acetylating PPARγ, thereby protecting its stability and influencing key metabolic pathways involved in hepatic lipid homeostasis.

Mitochondria possess approximately 1,200 proteins, but only 13 proteins are encoded by mitochondrial DNA. Nuclear-encoded proteins require several cytosolic factors to facilitate translocation of mitochondrial protein precursors to mitochondria from cytosol, thereby initiating mitochondrial import through the TOM-TIM system (12). This process probably requires normal mitochondrial membrane potential. GCN5L1 localization was independent of mitochondrial membrane potential loss (Supplemental Figure 1G), suggesting that the reduction in mitochondria-localized GCN5L1 under HFD or PA/OA treatment might be independent of the import process. Given the current lack of direct evidence for the protein import process, further isotope tracing of newly synthesized proteins and cell fractionation analysis are therefore necessary to confirm this mechanism. Meanwhile, a number of matrix proteins do not have a cleavable MTS, and the signals they possess are poorly studied, including GCN5L1. The emerging evidence presents new regulatory pathways that enable translocation of mitochondrial proteins containing MTS to cytosol or nucleus in response to mitochondrial or cellular stress (9–11, 20). These processes somehow rely on mitochondrial dysfunction, which activates IMS to remove the MTS from the precursors, initiating mitochondrial integrated stress response (ISR). However, the translocation of GCN5L1 is not related to mitochondrial ISR, as mitochondrial GCN5L1-to-cytosol translocation was not initiated by the treatment of mitochondrial inhibitors (FCCP and oligomycin) in primary hepatocytes (Supplemental Figure 1G), whereas PA/OA incubation promoted GCN5L1 translocation (Figure 1, C and D). PA/OA incubation promoted mPTP opening, which facilitates release of GCN5L1 by mitochondria (Supplemental Figure 1H). This demonstrates a mitochondrial retrograde mechanism for communicating with extra-mitochondrial signals.

PPARγ is a well-established key transcriptional factor in lipid synthesis within adipose tissues, given its critical role in adipocyte development and adipogenesis. However, its function in the liver is controversial. PPARγ enhances insulin sensitivity by upregulating PI3K and GLUT4, which promotes glucose uptake in adipose tissues and contributes to obesity (42). In parallel, PPARγ inhibits liver inflammation by regulating key inflammatory factors such as NF-κB and STAT, thereby alleviating hepatic metabolic disorders (32). Currently, PPARγ or pan-PPAR agonists are being investigated in clinical trials for the treatment of diabetes and MASH, with a focus on reducing liver inflammation and fibrosis (31). The role of PPARγ in hepatic lipid metabolism has been partially elucidated through studies showing that liver-specific deletion of PPARγ in mice fed a high-fat diet (HFD) impacts liver steatosis (34). Nevertheless, the understanding of its function in the liver remains incomplete, potentially because of the low transcriptional levels of PPARγ in normal livers. In adipocytes, E3 ligases such as TRIM25 and MKRN1 mediate PPARγ ubiquitination and degradation (43, 44). In adipose tissue, Sirt1 regulates PPARγ acetylation at K268 and K293, and deacetylation mutants have been shown to protect mice from HFD-induced obesity (45, 46). However, the regulatory pathways of PPARγ in the liver have remained elusive. In our current study, we discovered increased PPARγ expression in the livers of both MASLD patients and mouse models. Notably, the levels of PPARγ protein and hepatic triglyceride (TG) were correlated with cytoplasmic GCN5L1 levels. These findings uncover a regulatory pathway of PPARγ in the liver, shedding new light on the complex interplay between these factors in hepatic lipid metabolism and MASLD pathogenesis.

Indeed, previous research reported elevated hepatic FAO in humans with MASLD (47). However, a subsequent study using isotope tracing techniques found no significant alterations in FAO or the pyruvate cycle in MASLD patients (48), highlighting the complexity and conflicting evidence regarding metabolic pathways in this disease. Moreover, de novo lipogenesis is considered a major contributor to fatty acid synthesis, converting glucose and fructose into TGs during MASLD progression (49, 50). In brown fat, mitochondria play a role in lipogenesis by supplying citrate, a key substrate for de novo lipogenesis, particularly in mitochondria surrounding lipid droplets (51). However, the lipid droplet–associated mitochondria promote FAO (52), but associate with the severity of MALSD (5), suggesting complex and tissue-specific function and regulatory pathway of mitochondrial activity and metabolism. Mitochondrial signaling transduction encompasses multiple mechanisms: Reactive oxygen species can trigger oxidative stress responses, while the AMP/ATP ratio activates energy-sensing pathways, such as those involving AMPK. Additionally, mitochondrial metabolites can act as donors or acceptors for genomic modifications. Although cytosolic chaperones have been shown to translocate to the intermembrane space in response to cellular stress, the mechanisms and significance of mitochondrial protein translocation remain poorly understood. The observation of mitochondrial GCN5L1 translocation during MASLD progression represents a potential pathway. This finding may define a new characteristic feature of MASLD, offering insights into the disease’s pathophysiology and potentially uncovering new therapeutic targets.

Overnutrition or MASLD has long been recognized to be linked to increased mitochondrial protein acetylation, which is attributed to reduced Sirt3 levels and elevated mitochondrial acetyl-CoA content (53, 54). However, emerging evidence has revealed that multiple factors affect mitochondrial protein acetylation, e.g., nutrient types, duration of nutrient exposure, and fasting status (55, 56). GCN5L1 shares high homology with the cytosolic and nuclear acetyltransferase GCN5; however, it lacks the acetyltransferase catalytic domain while possessing an acetyl-CoA binding domain (57). Our current work demonstrates a significant correlation between GCN5L1 expression and protein acetylation, suggesting the crucial role of GCN5L1 in regulating protein acetylation (58). In vitro assay demonstrates that GCN5L1 can directly promote PPARγ acetylation (Supplemental Figure 6A), suggesting that GCN5L1 may increase local acetyl-CoA levels around its partners to facilitate a non-enzymatic-like acetylation. Since acetylation is a dynamically reversible process, identifying the deacetylases acting on PPARγ and elucidating their functions in normal liver metabolism represent compelling research directions. The NAD^+^/NADH ratio is known to regulate the cytosolic SIRT family of deacetylases. In our prior study (59), we found that GPD2, a key component of the mitochondrial NADH shuttle, is downregulated in GCN5L1-LKO livers and primary hepatocytes. This downregulation decreases the NAD^+^/NADH ratio, subsequently impairing sirtuin activity (especially Sirt1) by reducing the cytoplasmic NAD^+^ levels. Notably, the decrease in mitochondria-localized GCN5L1 during MASLD may disrupt the activity of the NADH shuttle. Such disruption could elevate mitochondrial NAD^+^ levels, potentially activating SIRT3 and contributing to the observed decrease in mitochondrial acetylation in MASLD livers.

SCD1 is the rate-limiting enzyme in the synthesis of unsaturated fatty acids, particularly oleate, the most abundant unsaturated fatty acid in the human body. In GCN5L1-LKO livers, SCD1 expression is decreased by more than 80%, indicating a predominant role of GCN5L1 in regulating SCD1 expression. We hypothesized that SCD1 might play a crucial role in lipid homeostasis in GCN5L1-LKO livers. Unexpectedly, overexpression of SCD1 failed to restore hepatic TG contents in GCN5L1-LKO livers. Intriguingly, SCD1 expression could alleviate liver injury in GCN5L1-LKO livers, suggesting that unsaturated fatty acids are essential for preventing liver injury in MASLD. The lipotoxicity of excess palmitic acid (PA) could induce liver injury. When hepatic PA levels are elevated, owing to either adipose tissue lipolysis or hepatic de novo lipogenesis, the synthesis of unsaturated fatty acids may mitigate the toxic effects of PA, facilitating the storage of TG in the liver. This could represent a compensatory pathway to mitigate the harmful effects of lipotoxicity. Our previous study indicated CISD1 (also known as MitoNEET), a mitochondrial iron transporter, as a substrate of GCN5L1 (60). GCN5L1 modulates its acetylation and activity to maintain mitochondrial iron homeostasis; modulating iron homeostasis also alleviates liver injury in GCN5L1-LKO mice. Moreover, iron levels were increased in GCN5L1-LKO livers under either normal chow or high-fat diet, suggesting that iron overload could be another reason for liver injury in GCN5L1-LKO mice.

## Methods

The materials and primers used in this study are included in Supplemental Tables 2 and 3. The detailed methods are given in Supplemental Methods.

### Sex as a biological variable.

This study used male mice as experimental subjects and did not consider sex as a biological variable. Male mice responded more effectively to high-fat diets and high-fat/high-cholesterol plus high-fructose (HFF) diets compared with female mice.

### Statistics.

All the in vivo animal experiments were randomized by genotype, and the investigators were not blinded to allocation during experiments and outcome assessment. All in vitro cell experiments were not randomized. There was no predetermination of sample size, and sample size was chosen based on available animal or cell numbers or clinical samples. Statistical analyses were performed using GraphPad Prism (v8.0) and SPSS (27.0). All experiments were performed at least 3 times independently. Data distribution was assumed to be normal without formal testing. For comparison between 2 groups in which *n* was 3 or 4, datasets were analyzed by a non-parametric test. For comparison between 2 groups in which *n* was greater than 4, datasets were analyzed by a 2-tailed Student’s *t* test. When the number of groups exceeded 2 and both normal distribution and homogeneity of variance were satisfied, a 1-way analysis of variance (ANOVA) with Bonferroni correction was used. When the number of groups exceeded 2 and normal distribution was satisfied but homogeneity of variance was not, Welch’s ANOVA with Games-Howell post hoc tests was used. For experiments with a 2-factorial design, multiple comparisons were analyzed by 2-way ANOVA to determine the statistical significance between groups based on 1 variable. *P* values of less than 0.05 were considered significant.

### Study approval.

Liver and clinical information was obtained from the Department of General Surgery, Tianjin Medical University General Hospital. This study was approved by the Ethics Committee of Tianjin Medical University General Hospital (IRB2025-YX-255-01). All animal protocols were in accordance with institutional guidelines and were approved by the Animal Care and Use Committee of Tianjin Medical University (TMUaMEC 2024049). The cell culture study was approved by the Department of Pharmacology, Tianjin Key Laboratory of Inflammatory Biology, School of Basic Medical Sciences, Tianjin Medical University.

### Data availability.

The experimental data are available upon request. RNA sequencing data were deposited in the BioProject database with accession number PRJNA1346149. Values for all data points in graphs are reported in the Supporting Data Values file.

## Author contributions

This study was conceived by LW and LZ. JZ, DW, QT, YC, LW, and LZ contributed to the study conception and design. Experiments were performed by JZ, DW, QT, YY, XL, XH, ZW, and LS. Human clinic samples were collected and provided by YH, JC, and LS. Data were analyzed and interpreted by JZ, DW, QT, LW, and LZ. The proteomic experiments were conducted, and the data analyzed, by XB and KZ. The manuscript was written by JZ, LW, and LZ with input from all authors.

## Funding support

- National Natural Science Foundation of China (grants 82322013 and 81970674 to LZ).
- National Key Research and Development Program of China (2024YFA1802704 to LZ).
- Natural Science Foundation of Tianjin, China (grants 23JCZDJC00530, 24ZXZSSS00030, and 24ZXZSSS00430 to LZ; grant 25JCQNJC00690 to ZW).
- Science & Technology Development Fund of the Tianjin Education Commission for Higher Education (grant 2024ZD038 to LZ).

## Supplementary Material

Supplemental data

Unedited blot and gel images

Supporting data values

## Version 1 01/23/2026

Electronic publication
