# Supplementary material for: Mitochondrial retrograde signal through GCN5L1 transition–mediated PPARγ stabilization promotes MASLD development
Source: JCI Insight. 2026 Jan 23;11(2):e196695. doi: 10.1172/jci.insight.196695 (PMC12892901; doi:10.1172/jci.insight.196695)
Supplement: Supplemental data [file jciinsight-11-196695-s059.pdf]

Supplementary Methods

Mouse studies

All mice were kept under isolated ventilated cages in an animal barrier facility at the Tianjin Medical University. The mice were maintained on a 12/12 light/dark cycle and housed with free access to water and pellet food. GCN5L1 flox/flox mice (provided by Dr. Michael Sack, NIH) were bred with Albumin-promoter-driven Cre (JAX) transgenic mice to generate liver tissue-specific GCN5L1 LKO mice. All mice were on a C57BL/6J background, and all experiments were performed using littermates.

Six- to eight-week-old GCN5L1 flox/flox (Con) and GCN5L1 liver knockout (LKO) mice were fed a high-fat diet (HFD) (Research Diets, D12492) or normal chow diet (Jiangsu Xietong, 1010055) for 16 weeks to establish liver steatosis model. And a MASH model was established by feeding high-fat/high-cholesterol plus high fructose (HFF) (Research Diets, D17010102) diet for 20 weeks.

Hepatic triglyceride extraction

Liver tissue was weighed on a balance and then homogenized in phosphate-buffered saline (PBS). The two-fold volume of chloroform and methanol (2:1 v/v) were mixed with liver homogenate and centrifuged to extract triglycerides and glycerol, and the lower liquid phase was transferred to a new EP tube. The air-dried triglyceride extract was then dissolved in ethanol containing 1% Triton X-100. The triglyceride levels were determined using a triglyceride assay kit (Sigma, T2449, F6428), and normalized to the protein concentrations.

Plasma parameters measurement

Blood was collected into an anticoagulation tube and centrifuged to obtain plasma. Plasma triglycerides (Sigma, T2449, F6428), cholesterol (Nanjing Jiancheng Bioengineering Institute A111-1-1), aspartate aminotransferase (AST) (Nanjing Jiancheng Bioengineering Institute C010-2-1), and alanine aminotransferase (ALT) Nanjing Jiancheng Bioengineering Institute C009-2-1) levels were then measured according to manufacturer instruction.

Oral Lipid Tolerance Test

Mice were starved for 4 hours and then received 0.2 ml olive oil by oral gavage. Blood was taken from the tail vein right before and at different time points after the oil load (0 min, 1 hr and 2 hr) to measure triglyceride levels in the plasma with a colorimetric assay kit (Sigma, T2449, F6428).

VLDL-TG Secretion

Mice were fasted for 6 hr. In vivo secretion rates of triglyceride (TG) into plasma were determined in unrestrained mice, following intraperitoneal injection of 1 g/kg body weight Poloxamer 407 (P-407, Sigma, 16758). Immediately before, at 60 min, and 120 min after P-407 administration, tail vein blood samples were taken for TG level measurement with a colorimetric assay kit (Sigma, T2449, F6428). TG secretion rates were calculated using the slope between 60 min and 120 min, based on the assumption of a plasma volume of 3.5% of body weight.

Histological analysis

4% PFA-fixed liver tissues, paraffin-embedded liver sections were stained with H&E and Sirius Red, and OCT compound-embedded frozen liver sections were stained with Oil red O.

Fatty acid uptake assays

The in vivo fatty acid uptake assay was previously described (1). Mice were starved for 6h and intraperitoneally injected 50μg BODIPY FL-C16 (ThermoFisher, D3821), 1 hour later, the mice were euthanized. The plasma, liver, gastrocnemius and subcutaneous WAT were collected for fluorescence detection and normalized to protein concentrations. Primary hepatocytes were incubated with BODIPY FL-C16 1μM in PBS with 20 μM FFA-free BSA (Sigma, A8806)) for 10 min in a 37°C incubator. Then the cells were washed with PBS, trypsinized and followed by flow cytometry analysis (Excitation 505nm and Emission 512nm).

Cell culture studies

Primary hepatocytes were isolated from CON or GCN5L1 LKO mice at age of 8-12 weeks following the previous protocol (14). Hepatocytes (6×10^5 per well, six-well plate) were seeded onto plates coated with collagen I (Sigma C3867) and cultured in DMEM medium containing 10% FBS and 1% penicillin/streptomycin. HepG2, AML12, and HEK293T cells were obtained from the American Type Culture Collection (ATCC). All cells were maintained in DMEM supplemented with 10% fetal bovine serum (FBS) and 1% penicillin/streptomycin and cultured at 37 degrees Celsius in an incubator containing 5% CO_2.

Western blot analysis

All samples were lysed with RIPA lysate (NCM, WB3100) containing protease inhibitor cocktail, centrifuged at 12,000 rpm and the supernatant was collected. Protein concentration was determined by BCA kit (CWBIO, CW0014S), the remaining protein was added to 5 × SDS PAGE sample buffer (GenStar, ZE153-101s). 50-80ug of protein was separated on an SDS-PAGE gel and transferred to a PVDF membrane. 10% milk was blocked at room temperature for 2 hours before overnight incubation of primary antibody. The next day, the

secondary antibody was incubated for 2 hours at room temperature and the results were visualized using a Super ECL Detection Reagent (Yeasen, 36208ES60).

Real-time Quantitative PCR

TransZol up kit (TRAN, ET111-01-V2) was used to extract RNA from samples. Complementary DNA was generated from 1 μg RNA using Hifair® III 1st Strand cDNA Synthesis SuperMix (Yeasen, 11141). mRNA level was assessed by real-time PCR using the QuantStudio real-time PCR system and SYBR Green PCR master mix (Yeasen). Relative gene expression was calculated with $2^{-\Delta\Delta C}$ method and normalized to Actin expression. The primers used in this study are listed in Supplementary Table 2.

RNA-seq, bioinformatics and KEGG analysis

RNA extractions from livers of 16-week HFD fed GCN5L1 flox/flox and LKO mice were performed using TRIzol (Life technologies) according to the manufacturer's instructions. RNA was prepared from 4 mice per group, and RNA-seq was performed at Novogene with paired-end 150 bp reads (NovaSeq 6000 Sequencing System, Illumina). Gencode gene annotations version M18 and the mouse reference genome major release GRCm38 was derived from https://www.gencodegenes.org/. Dropseq tools v.1.1249 were used for mapping the raw sequencing data to the reference genome. The resulting UMI-filtered count matrix was imported into R v.3.4.4. Before differential expression analysis using Limma v.3.40.650, sample-specific weights were estimated and used as coefficients alongside the experimental groups as a covariate during model fitting with Voom. DEGs were identified when the p-adj was <0.05 and the $\log_2$fold change was > 0.5. GO term enrichment and KEGG enrichment analysis displayed significantly enriched biological processes and pathways when the p-adj was less than 0.05.

Immunoprecipitation

Samples are lysed with lysis buffer (20 mM Tris-HCl, pH 8.0, 137 mM NaCl, 1% Nonidet P-40, 2 mM EDTA) containing protease inhibitors and primary antibodies were incubated with samples for 24 hours. Antibody-protein mixtures were incubated with protein A/G-agarose beads (Yeasen, 36417ES08) at 4°C for 4 hours. Beads were washed 4-6 times with 800ul of Cold Wash Buffer (10 mM Tris-base, pH 7.4, 1 mM EDTA, 1 mM EGTA, pH 8.0, 150 mM NaCl, 1% Triton X-100) and resuspended in 1× loading Buffer for 5 min at 95°C before Western blot analysis.

Mass spectrometry analysis of PPARγ modification

PPARγ was immune-captured using anti-FLAG resin (Sigma). The proteins were digested into peptides by trypsin (Promega). Tryptic peptides were desalted by μ-C18 Ziptip. After

desalting, each tryptic digest was redissolved in HPLC buffer A (0.1% (v/v) formic acid in water) and injected into a nano-LC system (EASY-nLC 1200, Thermo Fisher Scientific). Each sample was separated by a C18 column (75μm inner-diameter × 15 cm, 3 μm C18) with a 50 min HPLC gradient at a flow rate of 300 nL/min. The HPLC gradient was as follows: 5% to 5% solvent B (0.1% formic acid in acetonitrile) in 5 min, 5% to 25% solvent B in 32 min, 25% to 38% solvent B in 8 min, 38% to 100% solvent B in 2 min and hold for 6 min at 100% solvent B. The HPLC elute was electrosprayed directly into an Orbitrap Eclipse mass spectrometer (Thermo Fisher Scientific). The source was operated at 2.2 kV. The mass spectrometric analysis was carried out in a data-dependent mode. For MS1 survey scan, automatic gain control (AGC) target is 4e5 and the resolution is 60,000. The MS2 spectra were acquired with 15000 resolution.

Raw data files were analysed using Thermo Proteome Discoverer 3.0 with an overall false discovery rate (FDR) for peptides of less than 1%. Peptide sequences were searched using trypsin specificity and allowing a maximum of two missed cleavages. Carbamidomethylation on cysteine was specified as fixed modification. Ubiquitination or acetylation on lysine, acetylation on N-terminal and Oxidation on methionine were set as variable modifications. Mass tolerances for precursor ions were set at ±10 ppm for precursor ions and ± 0.02 Da for MS/MS.

Luciferase reporter activity

Cells were transfected with the PPRE firefly luciferase-reporter constructs (Addgene #1015) and Renilla luciferase reporter vector. 36 hours post transfection, luciferase expression was determined by measuring luminescence with the Dual-Luciferase Reporter Assay System (Promega, E1910). The firefly luciferase activity was normalized to Renilla luciferase activity.

In vivo metabolic measurements

Mice were housed individually in metabolic cages under a 12 h light/12 h dark cycle with free access to food and water. Mice were acclimatized in a metabolic cage for 1 d before the recording. Food intake, energy expenditure, physical activity, O_2 and CO_2 were assessed simultaneously. Data analysis of whole-body energy expenditures or oxygen consumption was conducted and corrected based on body-mass differences using a regression-based ANCOVA.

Lipidomic profiling and data analyses

All samples were collected and stored at −80 °C. Liver homogenization was resuspended in 300 μl methanol/water (v/v = 75:25), containing of LPC 18:1(d7) as the internal standard.

Then, each sample was sonicated for 30 s to ensure homogeneity and added with 750 µl MTBE, vortexed for 60 s, and gently vibrated for another 30 min. After that, the sample was added with 190 µl of nuclease-free water and vortexed for 1 min. After equilibration at room temperature for 10 min, the sample was centrifuged at 14,000g for 15 min. Then 400 µl aliquot of the upper lipid extract was pipetted into the new centrifuge tube and vacuum dried. The dried sample was dissolved with 150 µl of acetonitrile/isopropanol/water (v/v/v = 65:30:5) for the instrumental analysis in the positive and negative ion mode. The lipid profiling was acquired by a Q Exactive Plus high-resolution mass spectrometer (Thermo Fisher Scientific) equipped with an Ultimate 3000 UHPLC system (Thermo Fisher Scientific). Parameters on the chromatographic separation in the positive ion mode were the same as those in the negative ion mode. A total of 5 µl of the dissolved sample was injected for the lipid separation by a BEH C8 column (100 × 2.1 mm, 1.7 µm, Waters). The column temperature was 55 °C. The lipids were eluted by the binary mobile phase A (acetonitrile/water solution, v/v = 6:4, containing 10 mM ammonium acetate) and B (isopropanol/acetonitrile solution, v/v = 9:1, containing 10 mM ammonium acetate). The flow rate was 0.3 ml min−1. The elution gradient was conducted as follows: initial 32% B maintained for 1.5 min, linearly increased to 85% B from 1.5 to 12.5 min, to 97% B from 12.5 to 12.6 min, maintained at 97% B from 12.6 to 14.5 min, and then decreased to 32% B at 14.6 min, finally maintained at 32% B to 16.0 min. The temperature of the sample manager was 10 °C. The lipids eluted from the column were ionized by electrospray ionization in the mass spectrometer, and the mass signals were detected in full scan MS and data-dependent MS/MS (ddMS2) mode, with a resolution of 70,000 and 17,500, respectively. The spray voltage (kV) was +3.5 and −3.0 in the positive- and negative-ion mode, respectively. Other parameters were identical in the positive- and negative-ion mode, and set as follows: capillary temperature (°C), 300; aux gas heater temperature (°C), 350; sheath gas flow rate (a.u.), 45; aux gas flow rate (a.u.), 10; S-lens RF level, 50; mass scanning range (m/z), 150–1,500; TopN (N, the number of the fragmentation ions with the highest abundance), 10; stepped normalized collision energy, 25, 35 and 45%.Data processing was performed using MS-DIAL software.

Isolation of mitochondrial and cytoplasmic fractions

Tissues or cells were homogenized in isolation buffer (225 mM mannitol, 75 mM sucrose, 0.5% BSA, 0.5 mM EGTA and 30 mM Tris-HCl pH 7.4) and centrifuged at 800G for 5 minutes, the supernatant containing mitochondria was centrifuged again at 9,000 g for 10 min at 4 °C, and the sediment was collected containing mitochondrial fraction and supernatant was cytoplasmic fraction.

Site mutation assay

Mutant plasmid primers were designed by Quick change primer design website, and then

PCR was performed by Hieff Canace®II High-Fidelity DNA Polymerase. (Yeasen,10140ES60) The PCR products were co-incubated with DpnI (Beyotime, D6257) enzyme for 1hr to screen for mutant positive plasmids and then transformed in Top10 competent cells.

BODIPY staining

The fluorophore BODIPY 493/503 specifically stains neutral lipids was used. The cells were rinsed three times with PBS before staining with BODIPY (0.1ug/ml) for 10 min and counterstaining with DAPI for 10 min at 37℃ in the dark, according to the manufacturer's protocol. Images were captured under a confocal microscope.

Adeno-associated virus production

GCN5L1-Myc, Scd1, PPARγ-WT-Flag, PPARγ-K289R-Flag and eGFP were subcloned into AAV-TBG vector. AAVs were produced according to the protocol (2). First, HEK293T cells are transfected with three plasmids using PEI: (i) pAAV, which contains the rAAV genome of interest; (ii) pUCmini-iCAP-PHP, which encodes the viral replication and capsid proteins; and (iii) pHelper, which encodes adenoviral proteins necessary for replication. Using this triple-transfection approach, a single-stranded rAAV genome is packaged into an AAV-PHP capsid in HEK293T cells. AAV-PHP viruses are then harvested, purified, and tittered by quantitative PCR (qPCR). Purified viruses are intravenously delivered to mice via tail vein injection, and gene expression is later assessed using molecular, histological, or functional methods relevant to the experimental aims.

Generation of GCN5L1 knockout cell lines

HEK293T cells were co-transfected with lentiCRISPR-sgGCN5L1 plasmid and lentivirus packaging vectors using PEI (Polysciences, 24765-1), viruses were collected twice after 48 and 72 hours. HepG2 and AML12 cells were infected with appropriate amount of viruses, twenty-four hours after infection, the cells were selected with puromycin (1-2 mg/ml) for 2 weeks. Surviving colonies were then selected and expanded. The GCN5L1 knockout cells were then analyzed by Western blotting with a GCN5L1-specific antibody. The lentiviral sgGCN5L1 (sequence: GAGGTCCATACCCCACATTG) was generated as previously described (3).

In vitro acetylation assay

GCN5L1 protein was purified from BL21, Flag-tagged PPARγ was purified from 293T cells using anti-flag antibody. 10μg GCN5L1 was incubated in 30 μL reaction buffer (25 mM Tris-HCl, pH 7.4, 50 mM KCl and 1 mM dithiothreitol) with 1.5mM acetyl-CoA at 37°C for 3h, then samples were subjected for western blot analysis.

Quantification of mtDNA release

Hepatocytes (2 × 106 cells) were resuspended in 170 μl of digitonin buffer containing 150 mM NaCl,50 mM HEPES pH 7.4, and 25 μg/ml digitonin. The homogenates were incubated on a rotator for 10 min at room temperature, followed by centrifugation at 16,000 × g for 25 min at 4°C. A 1:20 dilution of the supernatant (cmtDNA) was used for qPCR. The pellet was resuspended in 340 ul of lysis buffer containing 5 mM EDTA and proteinase K and incubated at 55°C overnight. The digested pellet was diluted with water (1:20 to 1:100) and heated at 95°C for 20 min to inactivate proteinase K, and the sample was used for qPCR with mtDNA specific primers (Table. S2).

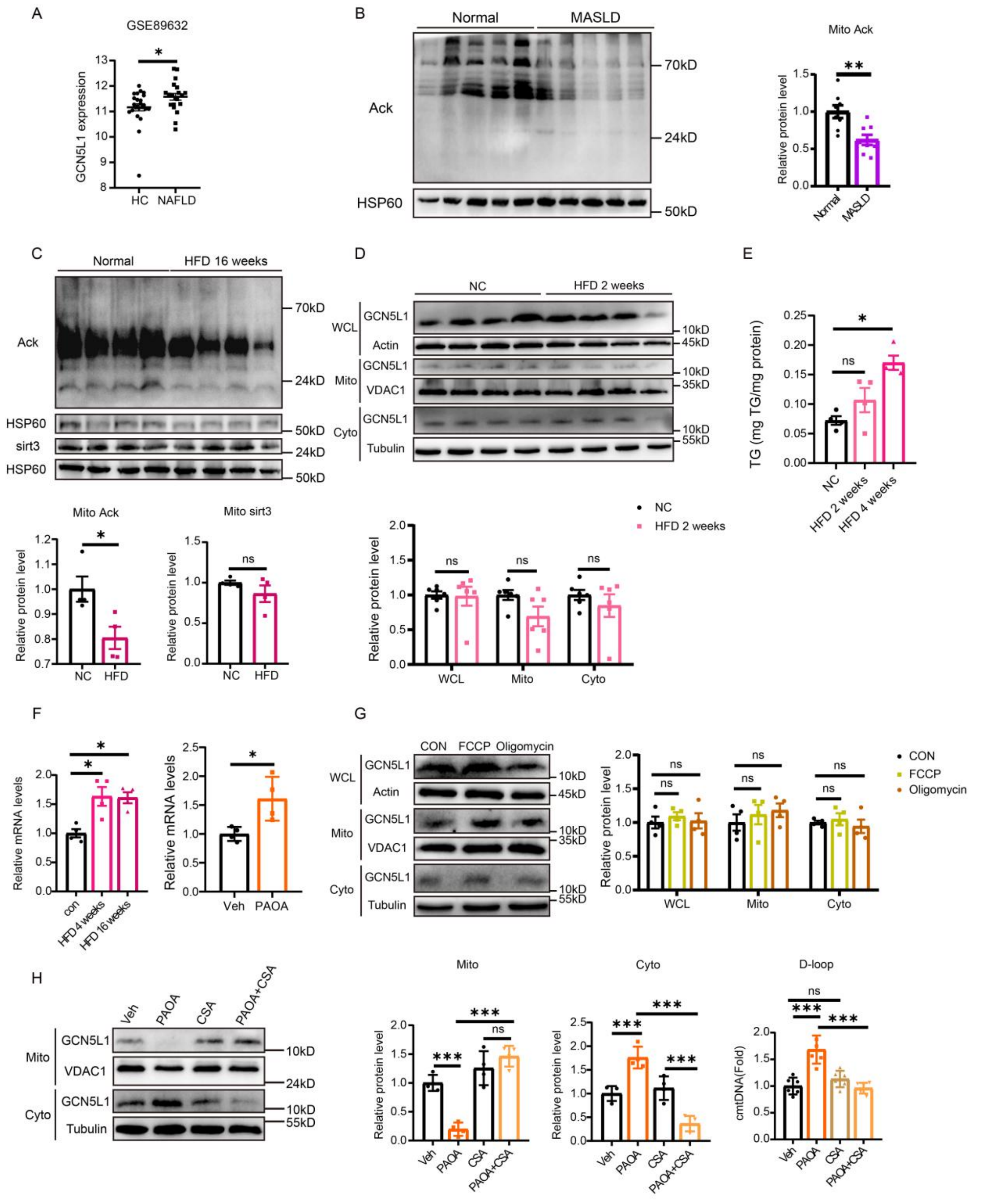

Supplementary Fig. 1 Related to Figure 1.

(A) GEO database analysis of GCN5L1 expression in healthy and NAFLD (MASLD) populations.

(B) Western blot analysis of mitochondrial acetylation levels in Normal or MASLD patients, n=8 for each group, corresponding to the patients in Figure 1A.

(C) Western blot analysis of mitochondrial protein acetylation levels and Sirt3 levels in mitochondrial fractions from mice on normal or high-fat diets for 16 weeks, n=4 mice/group, corresponding to the mice in Figure 1B.

(D) GCN5L1 protein levels and quantifications of WCL, Mito and Cyto fractions of liver samples from Normal chow (NC) or 2-weeks high-fat diet (HFD) fed mice, n=6 mice/group.

(E) Liver triglyceride (TG) contents were determined in mice maintained on a normal diet, a 2-weeks high-fat diet or a 4-weeks high-fat diet, n=4 mice/group.

(F) GCN5L1 RNA levels in the liver of mice fed with a normal diet, a 2-week high-fat diet or a 4-week high-fat diet (n=4 for each group, left panel), as well as in primary hepatocytes from wildtype mice with PAOA or vehicle treatment for 15 hours (right panel).

(G) GCN5L1 protein levels and quantifications of WCL, Mito and Cyto fractions of primary hepatocytes treated with FCCP (1 μM) and oligomycin (2.5 μM) overnight, n=4 independent experiments.

(H) Primary hepatocytes were isolated from wild-type mice and incubated with 0.4mM PA/0.8mM OA and CsA (5uM) or vehicle for 15 hours. Mitochondrial and cytoplasmic fractions were isolated and subjected to immunoblotting for GCN5L1 levels (left). Mitochondrial DNA (mtDNA) levels were quantified in cytoplasmic fractions using qPCR (right).

FigS1A and FigS1B datasets were analyzed by a two-tailed Student's t-test. FigS1C and FigS1F(right) datasets were analyzed by a nonparametric statistical tests. FigS1D datasets were analyzed by a two-way analysis of variance (ANOVA) with Bonferroni correction. FigS1E, FigS1F(left), FigS1G and FigS1H datasets were analyzed by a one-way analysis of variance (ANOVA) with Bonferroni correction. Significance was considered as n.s. $P > 0.05$, *$P < 0.05$, **$P < 0.01$; ***$P < 0.001$

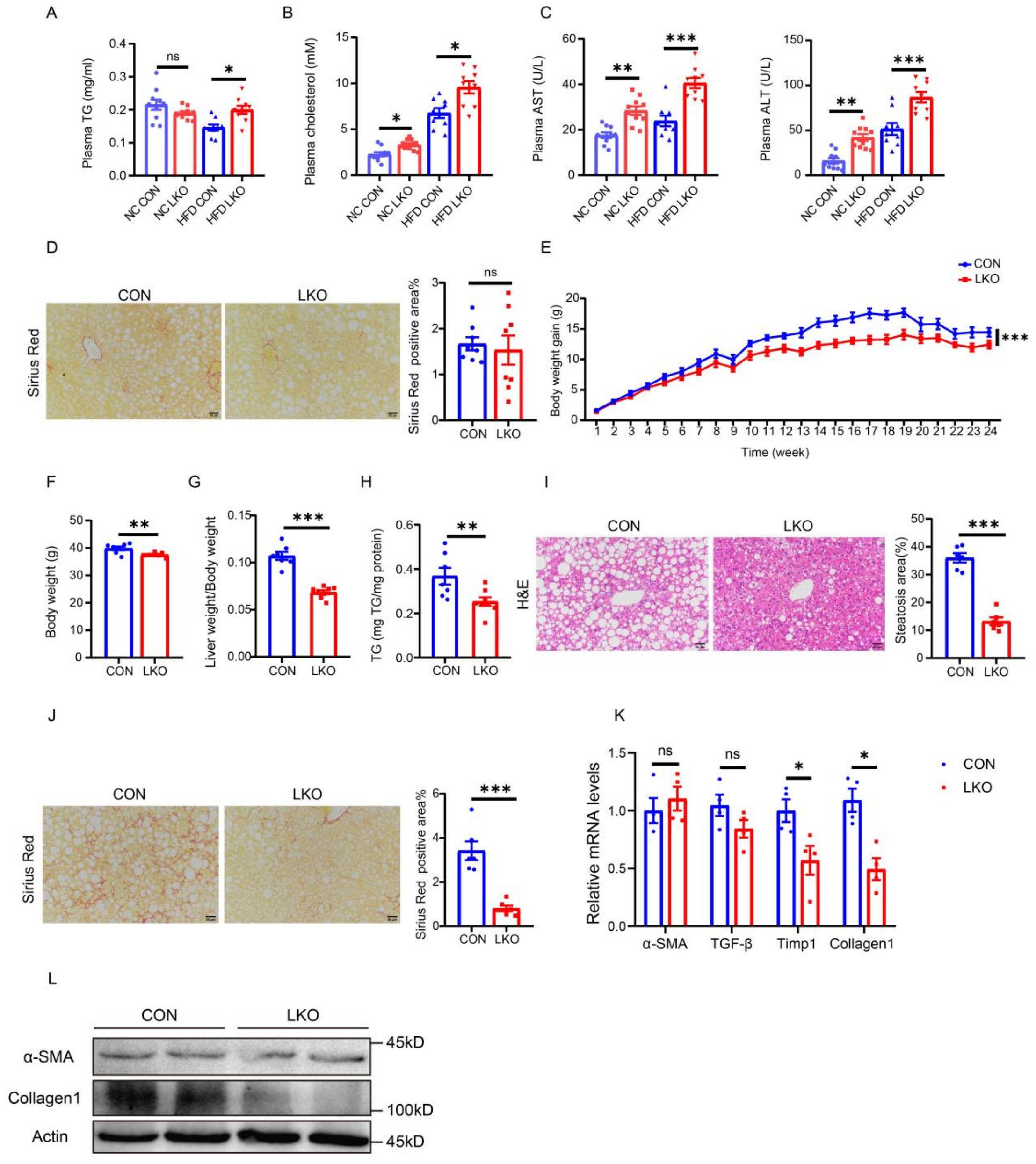

Supplementary Fig. 2. Related to Figure 2

(A) Plasma TG of CON or LKO mice on NC or HFD for 16 weeks. n=10 for NC, n=9 for HFD.

(B) Plasma cholesterol of CON or LKO mice on NC or HFD for 16 weeks. n=10 for NC, n=9 for HFD.

(C) Plasma AST and ALT of CON or LKO mice on NC or HFD for 16 weeks. n=10 for NC, n=9 for HFD.

(D) Representative images of Sirius Red staining of livers from CON or LKO mice on a HFD for 16 weeks. Scale bar=50μm, n=8 mice/group.

(E) Body weight gain of CON or LKO mice on a high-fat/high-cholesterol plus high fructose

(HFF) diets for 24 weeks, n=7 mice/group.

(F) Body weights of CON or LKO mice on a HFF diets for 24 weeks, n=7 for CON, n=8 for LKO.

(G) The ratio liver/body weight of CON or LKO mice on a HFF diets for 24 weeks, n=7 for CON, n=8 for LKO.

(H) Liver triglyceride (TG) contents of of CON or LKO mice on a HFF diets for 24 weeks, n=7 for CON, n=8 for LKO.

(I) Representative images of H&E staining of livers from CON or LKO mice on a HFF diets for 24 weeks. Scale bar=50μm, n=6 mice/group.

(J) Representative images of Sirius Red staining of livers from CON or LKO mice on a HFF diets for 24 weeks, n=6 mice/group.

(K) QPCR detection of fibrosis markers in livers from CON or LKO mice on a HFF diets for 24 weeks weeks, n=4 mice/group.

(L) Western blot analysis of fibrosis markers in CON or LKO mice on a HFF diet for 24 weeks.

FigS2A and FigS2C datasets were analyzed by one-way analysis of variance (ANOVA) with Bonferroni correction. FigS2B datasets were analyzed by a Welch's analysis of variance with Games-Howell post hoc tests. FigS2D, FigS2F, FigS2G, FigS2H, FigS2I and FigS2J datasets were analyzed by two-tailed Student's t-test. FigS2E datasets were analyzed by a two-factor repeated measures analysis of variance (ANOVA) with Bonferroni correction. FigS2K datasets were analyzed by a nonparametric statistical tests. Significance was considered as n.s. $P > 0.05$, $^*P < 0.05$, $^{**}P < 0.01$; $^{***}P < 0.001$.

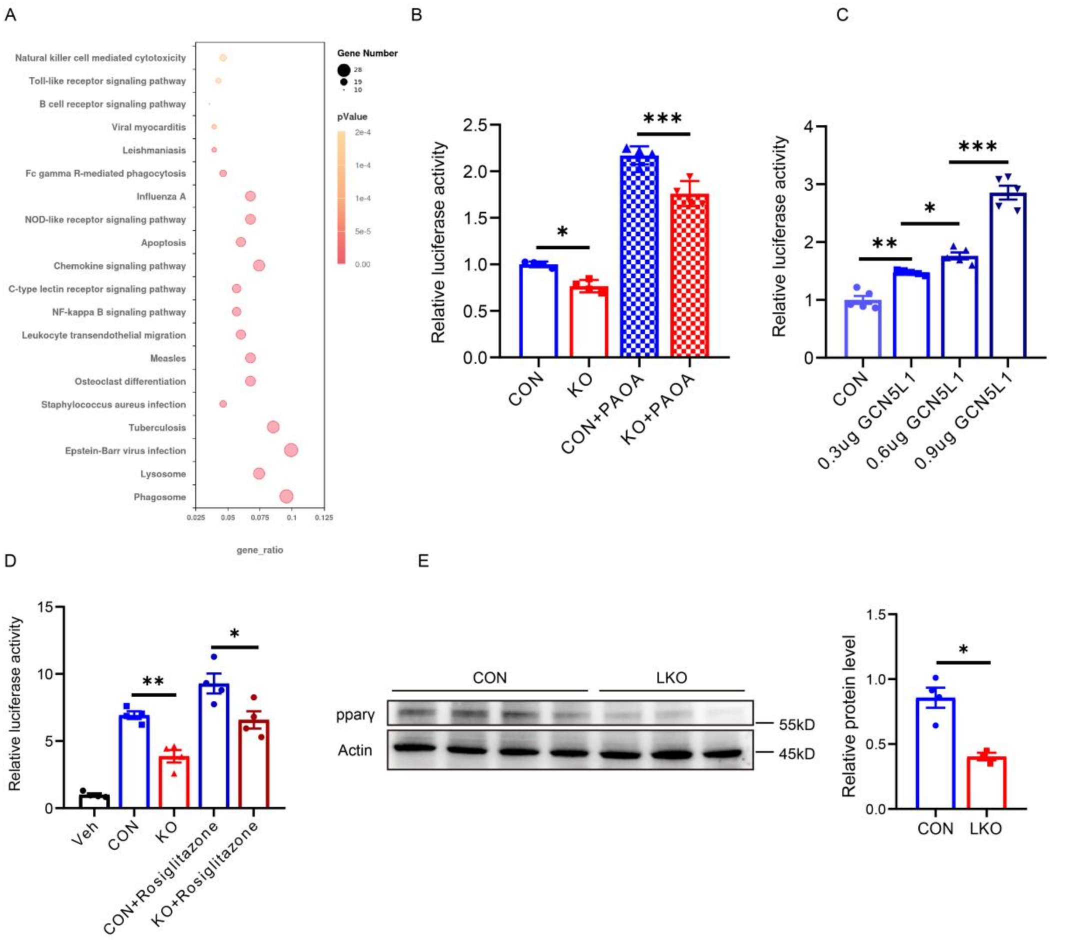

Supplementary Fig. 3. Related to Figure 4

(A) KEGG analysis of all up-regulated genes, analyzed by RNAseq.

(B) AML12 CON or KO cells were assayed for PPRE Luciferase activity after overnight stimulation with/without 0.1 mM PA/O.2 mM OA, n=4 for each group.

(C) Following overnight stimulation with 0.1 mM PA/0.2 mM OA, PPRE luciferase activity was detected in AML12 cells transfected with varying concentrations of GCN5L1, n=5 for each group.

(D) AML12 CON or KO cells were assayed for PPRE Luciferase activity after overnight stimulation with/without 1uM Rosiglitazone, n=4 for each group.

(E) PPARγ protein levels in hepatocytes from CON or LKO mice after overnight stimulation with 0.4 mM PA/0.8 mM OA, n=4 for CON, n=3 for LKO.

FigS3B and FigS3D datasets were analyzed by one-way analysis of variance (ANOVA) with Bonferroni correction. FigS3C datasets were analyzed by a Welch's analysis of variance with Games-Howell post hoc tests. FigS3E datasets were analyzed by a nonparametric statistical tests. Significance was considered as n.s. P > 0.05, *P < 0.05, **P < 0.01; ***P < 0.001.

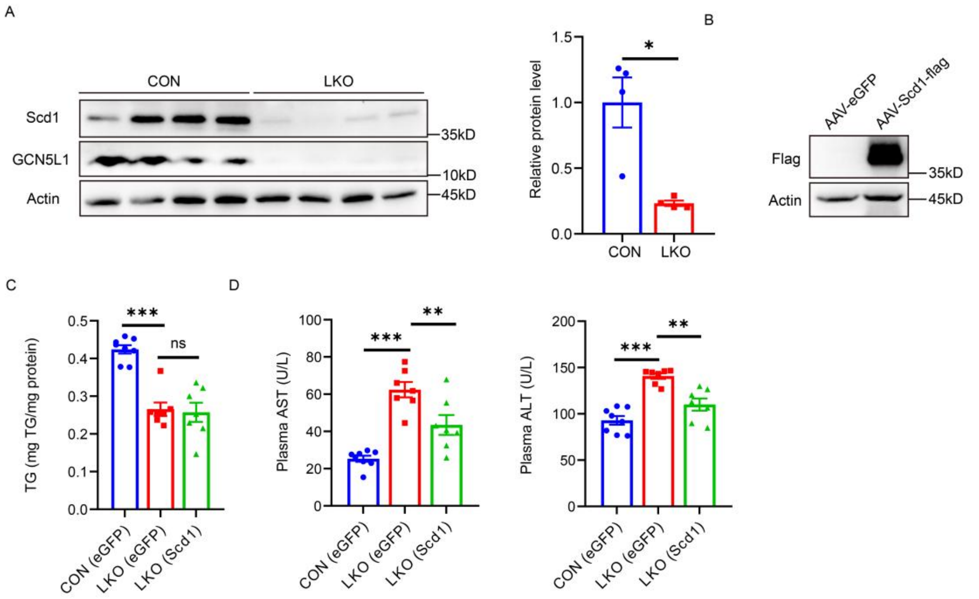

Supplementary Fig. 4. Related to Figure 4

(A) SCD1 protein levels in livers from CON or GCN5L1 LKO mice with HFD, n=4 mice/group.

(B) Western blot analysis was performed to validate the expression of the AAV-Scd1-flag virus.

(C) Liver triglyceride (TG) contents of CON-eGFP, LKO-eGFP or LKO-Scd1-flag mice mice on a HFD for 16 weeks, n=8 for CON with AAV-eGFP, n=7 for LKO with AAV-eGFP and LKO with AAV-Scd1-flag, CON mice are the same batch as in Figure 1H.

(D) Plasma AST and ALT of CON-eGFP, LKO-eGFP or LKO-Scd1-flag mice on a HFD for 16 weeks. n=8 for CON with AAV-eGFP, n=7 for LKO with AAV-eGFP and LKO with AAV-Scd1-flag.

FigS4A datasets were analyzed by a nonparametric statistical tests. FigS4C and FigS4D datasets were analyzed by a one-way analysis of variance (ANOVA) with Bonferroni correction. Significance was considered as n.s. $P > 0.05$, $^{*}P < 0.05$, $^{**}P < 0.01$; $^{***}P < 0.001$.

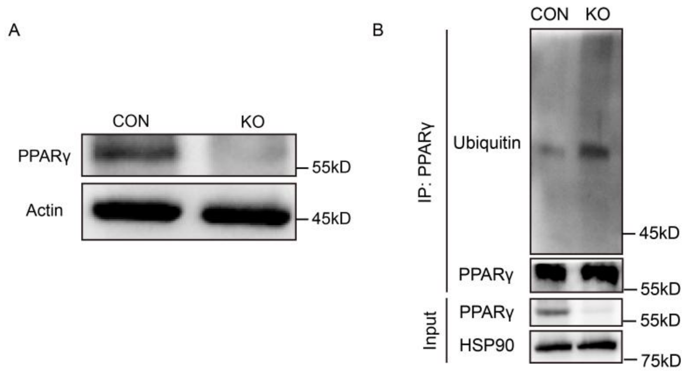

Supplementary Fig. 5. Related to Figure 5

(A) PPARγ protein levels in GCN5L1 KO and control C3H10T 1/2 cells.

(B) PPARγ antibody was utilized for immunoprecipitation of endogenous PPARγ, ubiquitination levels were analyzed by immunoblotting in GCN5L1 KO and control C3H10T 1/2 cells.

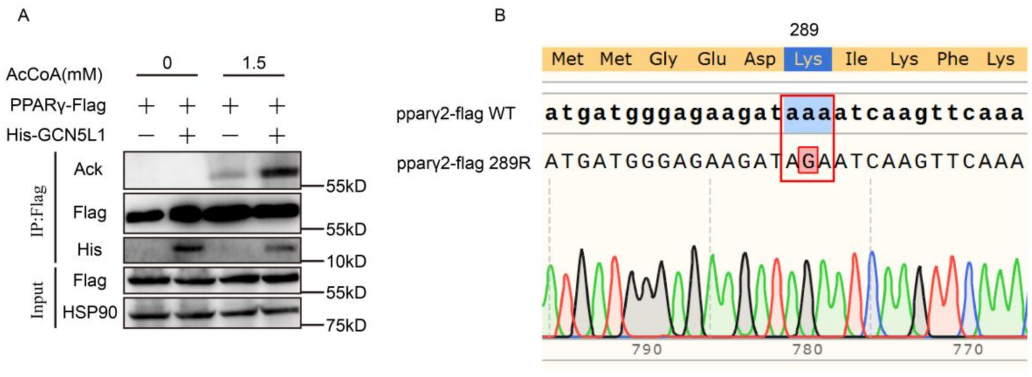

Supplementary Fig. 6. Related to Figure 6

(A) GCN5L1 was purified from BL21 cells. PPARγ was immunoprecipitated using flag resin from 293T cells expressing PPARγ-flag. GCN5L1 and PPARγ were incubated in the presence or absence of 1.5 mM acetyl-CoA, followed by immunoblot analysis to detect acetyl-PPARγ levels.

(B) Sequence of PPARγ 289R mutant.

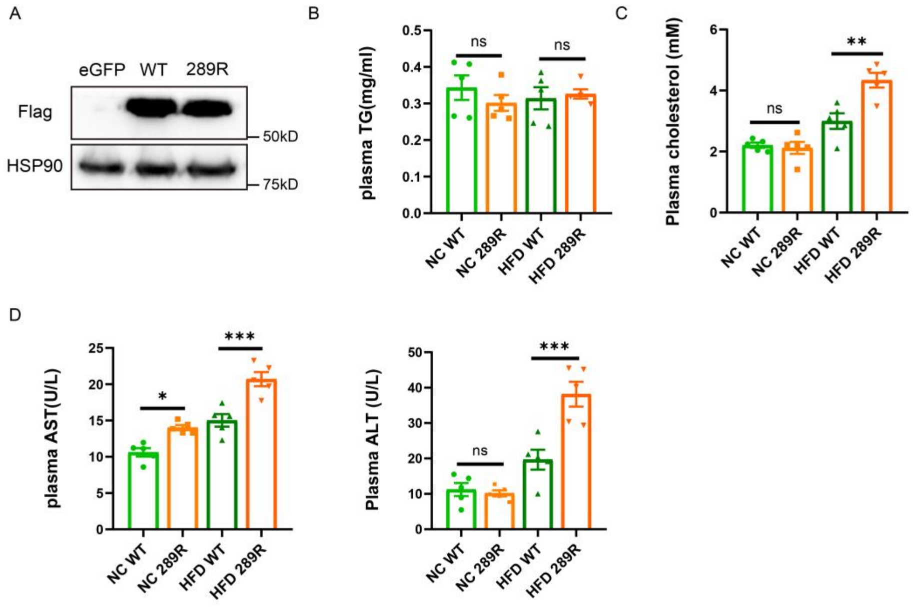

Supplementary Fig. 7. Related to Figure 7

(A) Western blot analysis was performed to validate the expression of the PPARγ-flag-K289R mutant plasmid.

(B) Plasma TG of PPARγ-flag WT or K289R mice on a HFD for 4 weeks, n=5 mice/group.

(C) Plasma cholesterol of PPARγ-flag WT or K289R mice on a HFD for 4 weeks, n=5 mice/group.

(D) Plasma AST and ALT of PPARγ-flag WT or K289R mice on a HFD for 4 weeks, n=5 mice/group.

FigS7B, FigS7C and FigS7D datasets were analyzed by a one-way analysis of variance (ANOVA) with Bonferroni correction. Significance was considered as n.s. $P > 0.05$, *$P < 0.05$, **$P < 0.01$; ***$P < 0.001$.

Table S1. Clinical Characteristics of Patients from Human Liver Biopsy Samples

	Normal (n=8)	MASLD (n=8)
Age	54.50 ± 4.52	35.25 ± 2.36[b]
Weight (kg)	72.31 ± 5.06	118.79 ± 6.66[c]
Height (m)	1.66 ± 0.04	1.72 ± 0.03
BMI (kg/m^2)	26.09 ± 0.76	40.15 ± 1.88[c]
Liver TG (mg TG/mg protein)	0.08 ± 0.01	0.30 ± 0.07[b]
Plasma AST (U/L)	22.50 ± 2.16	53.75 ± 17.41
Plasma ALT (U/L)	23.00 ± 2.36	70.13 ± 9.84[c]
Plasma TG(mM)	1.66 ± 0.30[d]	3.36 ± 0.52[a]
Plasma TC(mM)	2.14 ± 0.15[d]	3.61 ± 0.29[cd]
Plasma HDL(mM)	0.74 ± 0.07[d]	1.01 ± 0.07[a]
Plasma LDL(mM)	1.75 ± 0.33[d]	3.28 ± 0.30[b]
[a]P＜0.05 versus control		
[b]P＜0.01 versus control		
[c]P＜0.001 versus control		
[d]n=7		

All Data were performed using the paired, two-tailed Student's t-test.
All data are reported as mean ± SEM and values less than 0.05 were considered statistically significant

Table S2. Real-time PCR Primer sequences used in the experiments

Gene name (mouse)	Forward Primer	Reverse Primer
Actin	CCAGCCTTCCTTCTTGGGTA	CAATGCCTGGGTACATGGTG
Fasn	AGGTGGTGATAGCCGGTATGT	TGGGTAATCCATAGAGCCCAG
Scd1	GCGATACACTCTGGTGCTCA	CCCAGGGAAACCAGGATATT
Acc1	TGACAGACTGATCGCAGAGAAAG	TGGAGAGCCCCACACACA
Srebp1c	GGAGCCATGGATTGCACATT	GGCCCGGGAAGTCACTGT
Me	GCCGGCTCTATCCTCCTTTG	TTTGTATGCATCTTGCACAATCTTT
Gpat	AGCAAGTCCTGCGCTATCAT	CTCGTGTGGGTGATTGTGAC
Chrebp	CCTCACTTCACTGTGCCTCA	ACAGGGGTTGTTGTCTCTGG
Pparγ	CTGGCCTCCCTGATGAATAA	CGCAGGTTTTTGAGGAACTC
Srebp2	CCGCTCTCGAATCCTCTTAT	CAGCACCTGACTCCAGTGAC
Hmgcs2	GGTGTCCCGTCTAATGGAGA	ACACCCAGGATTCACAGAGG
CD36	TGGTCAAGCCAGCTAGAAA	CCCAGTCTCATTTAGCCAC
Fabp	GGGATGGAAAGTCGACCACA	CTTGTGGAAGTCACGCCTTT
Ppara	TCTGTGGGCTCACTGTTCTG	AACTACCTGCTCAGGGCTCA
Cpt1a	GCGAAGTGTCGGCAGACCTA	TGTTCCGATTCGTCCAACGT
ApoB	CGTGGGCTCCAGCATTCTA	TCACCAGTCATTTCTGCCTTTG
ApoE	GCTGGGTGCAGACGCTTT	TGCCGTCAGTTCTTGTGTGACT
GCN5L1	TCCCGCCTGCTCAAAGAAC	GAGGTGATCCACCAACGCTT
Cidea	GCAGCCTGCAGGAACTTATC	TCATGAAATGCGTGTTGTCC
Cidec	AAGATGGCACAATCGTGGAG	TTAGTTGGCTTCTGGGAAAGG
Mogat1	TGGCCAAGATCTGGTTCTGT	CCTTAGACACTGAAACCGGCCCGT
a-SMA	GTCCCAGACATCAGGGAGTAA	TCGGATACTTCAGCGTCAGGA
TGF-β	CTTCAATACGTCAGACATTCGGG	GTAACGCCAGGAATTGTTGCTA
Timp1	CTTGGTTCCCTGGCGTACTC	ACCTGATCCGTCCACAAACAG
Collagenl	TGGAAACCCGAGGTATGCTT	CATTGCATTGCACGTCATCG
D-loop	AATCTACCATCCTCCGTGAAACC	TCAGTTTAGCTACCCCCAAGTTTAA
16s	CACTGCCTGCCCAGTGA	ATACCGCGGCCGTTAAA

Table S3. Materials used in this study

REAGENT or RESOURCE	SOURCE	IDENTIFIER
Antibodies		
Rabbit polyclonal anti-GCN5L1	Gift from Dr. Michael Sack, NIH	N/A
PPARγ	Cell Signaling Technology	Cat# 2443
Acetylated-Lysine	Cell Signaling Technology	Cat# 9441
HSP90	Cell Signaling Technology	Cat# 4847S
Rabbit (DA1E) mAb IgG XP® Isotype Control	Cell Signaling Technology	Cat# 3900S
HSP60	Santa Cruz	sc-59567
Actin	ABClonal	Cat# AC026
Tubulin	ABClonal	Cat# AC012
VDAC1	ABClonal	Cat# A19707
Myc	ABClonal	Cat# AE070
Flag	Sigma-Aldrich	Cat# F1804
Ubiquitin	Proteintech	Cat# 10201-2-AP
Alpha smooth muscle actin	Proteintech	Cat# 67735-1-lg
Collagen Type I	Proteintech	Cat#1 14695-1-AP
Bacterial and virus strains		
TOP10 competent cells	Yeasen	Cat# 11801ES80
H128		
pLenti-EF1a-EGFP-3FLAG-PGK-Puro	OBiO Technology	N/A
LentiCRISPRv2	OBiO Technology	N/A
Chemicals and recombinant proteins		
NCM RIPA buffer	NCM Biotech	Cat# WB3100
PMSF	Solarbio	Cat# P8340
protease Inhibitor Cocktail	MedChemExpress	Cat# HY-K0010
TSA	Solarbio	Cat# IT1250
Phosphatase Inhibitor Cocktail	MedChemExpress	Cat# HY-K0021
Cycloheximide	MedChemExpress	Cat# HY-12320
Bafilomycin A1	MedChemExpress	Cat# HY-100558
FCCP	MedChemExpress	Cat# C2920
Oligomycin	MedChemExpress	Cat# 495455
Digitonin	MedChemExpress	Cat# HY-N4000
Recombinate DNase I	TaKaRa	Cat#2270A
Benzonase	Sigma-Aldrich	Cat# E1014-25KU

MG132	Beyotime	Cat# S1748-5mg
Cyclosporin A	Beyotime	Cat# S1563
DpnI	Beyotime	Cat# D6257
DMSO	Solarbio	Cat# 67-68-5
Bovine Serum Albumin V	Solarbio	Cat# A8020
TRIzol	Thermo Fisher	Cat# 15596018
rProtein A/G MagBeads (IP Grade)	Yeasen	Cat# 36417ES08
BODIPY™ 493/503	Thermo Fisher	Cat# D3922
BODIPY™ FL C_{16}	Thermo Fisher	Cat# D3821
Hieff TransTM Liposomal Transfection Reagent	Yeasen	Cat# 40802ES03
PEI	Polysciences	Cat# 24765-1
RNAiMAX	Thermo Fisher	Cat# 13778150
TritonX-100	Sangon Biotech	Cat# A110694
DMEM	Meilunbio	Cat# MA0212
FBS	ExCell	Cat# FSP500
Puromycin	Solarbio	Cat# P8230
Collagen Type1	Sigma-Aldrich	Cat# C3867
RNase A	Yeasen	Cat# 10405ES03
proteinase K	Solarbio	Cat# P9460
oleic acid	Sigma-Aldrich	Cat# O1008
palmitic acid	Sigma-Aldrich	Cat# P0500-10G
Poloxamer 407	Sigma-Aldrich	Cat# 16758-250G
Rosiglitazone	Sigma-Aldrich	Cat# 557366-M
olive oil	Aladdin	Cat# O108686-100ml
Biological samples		
Liver and blood biochemical tests of subjects	Tianjin Medical University General Hospital	Related to Figure 1A, S1B, TableS1
Critical commercial assays		
PurePlasmid Mini Kit	CWBIO	Cat# CW0500M
Gel Extraction Kit	Omega	Cat# D2500
Hieff Canace® Gold High Fidelity DNA Polymerase	Yeasen	Cat# 10148ES60
Hieff Clone® Plus Multi One Step Cloning Kit	Yeasen	Cat# 10912
Hifair® Ⅲ 1st Strand cDNA Synthesis SuperMix for qPCR	Yeasen	Cat# 11141

Hieff® qPCR SYBR Green Master Mix	Yeasen	Cat# 1120IES03
Free Glycerol Reagent	Sigma-Aldrich	Cat# F6428
Serum Triglyceride Determination Kit	Sigma-Aldrich	Cat# TR0100
Aspartate aminotransferase Assay Kit	Nanjing Jiancheng	Cat# C010-2-1
Alanine aminotransferase Assay Kit	Nanjing Jiancheng	Cat# C009-2-1
Total cholesterol assay kit	Nanjing Jiancheng	Cat# A111-1-1
Dual-Luciferase Reporter Assay System	Promega	Cat# E1910
Experimental models: Cell lines		
C3H10T 1/2	Gift from Feifan Guo	N/A
293T	ATCC	Cat# CRL-3216
AML12	ATCC	Cat# CRL-2254
HepG2	ATCC	Cat# HB-8065
C3H10T 1/2 GCN5L1-KO	This paper	N/A
HepG2 GCN5L1 KO	This paper	N/A
Experimental models: Organisms/strains		
Mouse: WT: C57BL/6J	Shanghai Laboratory Animal Center (SLAC)	N/A
Mouse: GCN5L1 f/f (flox)	Michael Sack lab	N/A
Mouse: Albumin-Cre	The Jackson Lab	jax.org/strain/003574
Recombinant DNA		
pLenti-GCN5L1-Flag	This paper	N/A
pLenti-GCN5L1-Myc	This paper	N/A
pLenti-PPARγ-Flag	This paper	N/A
pLenti-PPARγ-K289R-Flag	This paper	N/A
Ubiquitin-Myc	This paper	N/A
AAV-PPARγ-Flag	This paper	N/A
AAV-PPARγ-K289R-Flag	This paper	N/A
AAV-GCN5L1-Myc	This paper	N/A
AAV-Scd1-Myc	This paper	N/A
Software and algorithms		
GraphPad Prism 8.0	Graphpad Software	https://www.graphpad-prism.cn/
ImageJ 1.53k	ImageJ	https://imagej.nih.gov/ij/

Image-Pro Plus 6.0	Image-Pro Plus	https://mediacy.com/image-pro/
ZEN 2.3 (blue edition)	ZEISS	https://www.zeiss.com.cn/corporate/home.html
SnapGene 5.2	SnapGene	https://www.snapgene.cn/
Adobe Illustrator 27.5	Adobe	https://www.adobe.com/cn/products/illustrator.html
Other		
Normal Chow Diet (NCD)	Jiangsu Xietong	Cat# 1010055
High-fat/high-cholesterol plus high fructose diet (HFF)	Research Diets	Cat# D17010102
High-fat diet (HFD)	Research Diets	Cat# D12492
Direct-load Color Pertained Protein Marker	GenStar	Cat# M222
GoldBand 15000 DNA Marker	Yeasen	Cat# 10512ES
GoldBand 100 bp DNA ladder	Yeasen	Cat# 10507ES

Reference

1. Pacella I, Procaccini C, Focaccetti C, Miacci S, Timperi E, Faicchia D, et al. Fatty acid metabolism complements glycolysis in the selective regulatory T cell expansion during tumor growth. *Proc Natl Acad Sci U S A.* 2018;115(28):E6546-E55.
2. Grieger JC, Choi VW, and Samulski RJ. Production and characterization of adeno-associated viral vectors. *Nat Protoc.* 2006;1(3):1412-28.
3. Zhang T, Cui Y, Wu Y, Meng J, Han L, Zhang J, et al. Mitochondrial GCN5L1 regulates glutaminase acetylation and hepatocellular carcinoma. *Clin Transl Med.* 2022;12(5):e852.
