# Supplementary material for: Mitochondrial retrograde signal through GCN5L1 transition–mediated PPARγ stabilization promotes MASLD development
Source: JCI Insight. 2026 Jan 23;11(2):e196695. doi: 10.1172/jci.insight.196695 (PMC12892901; doi:10.1172/jci.insight.196695)

Full unedited gel for Figure 1A

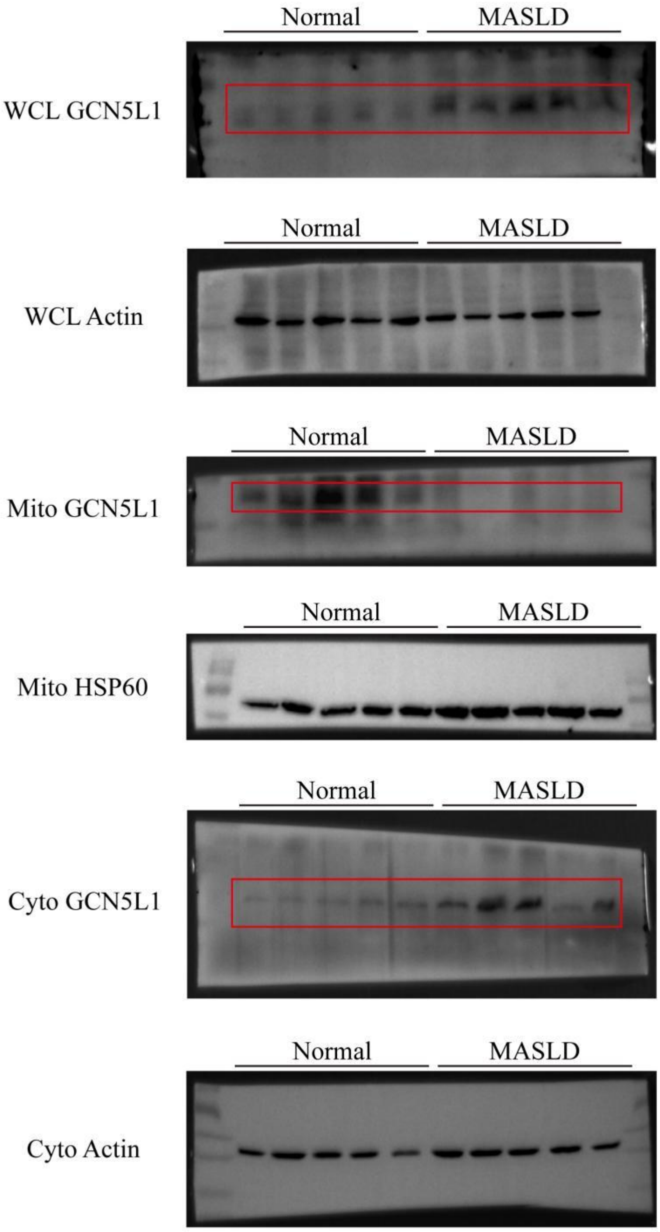

Full unedited gel for Figure 1B

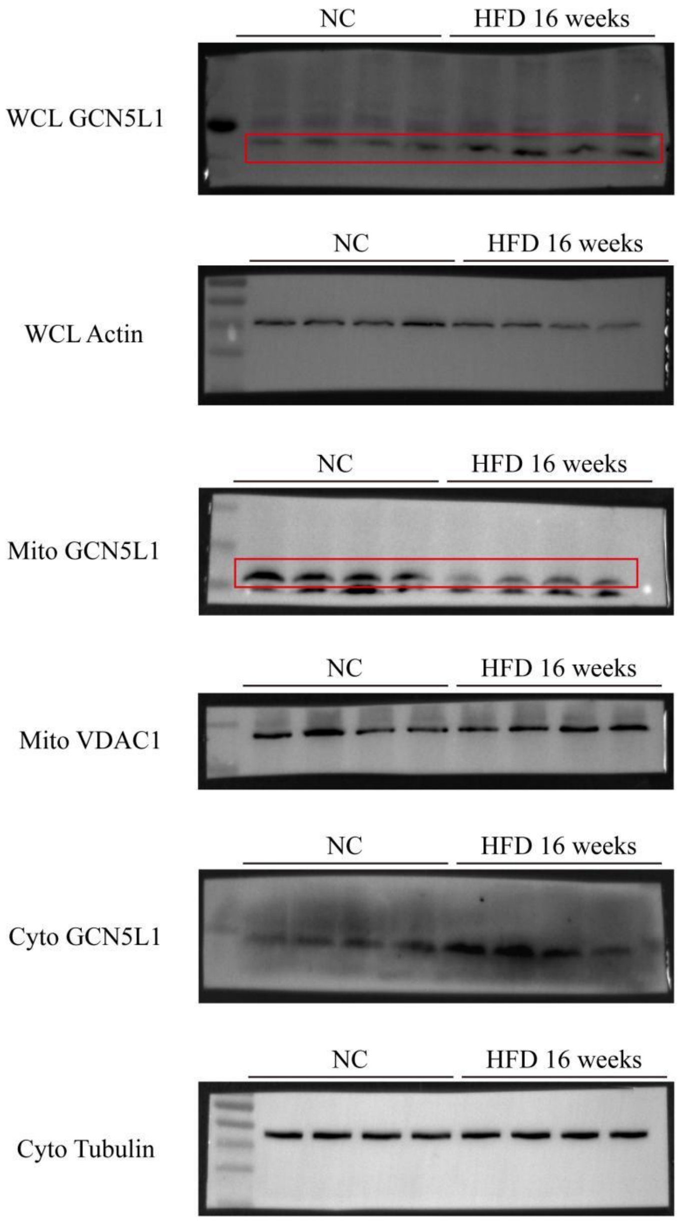

Full unedited gel for Figure 1D

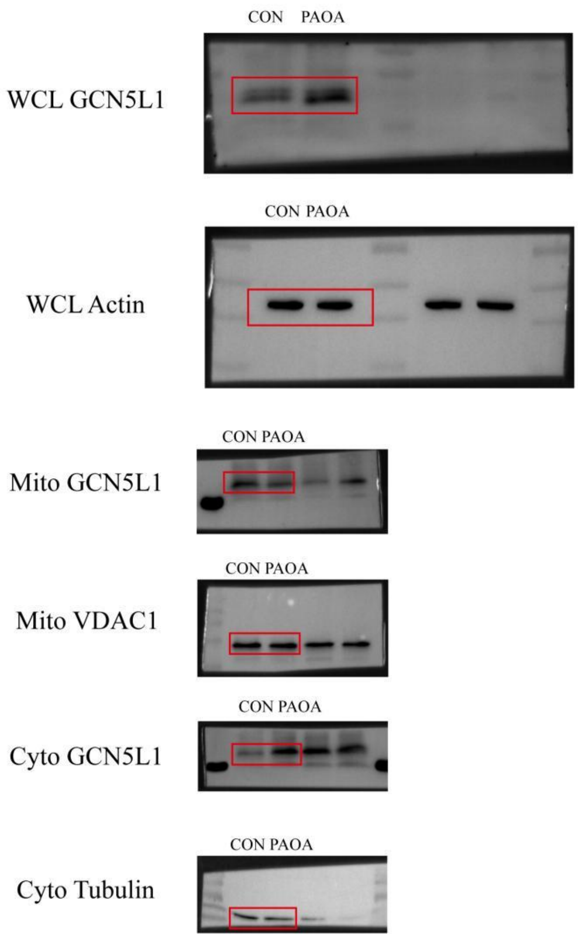

Full unedited gel for Figure 1E

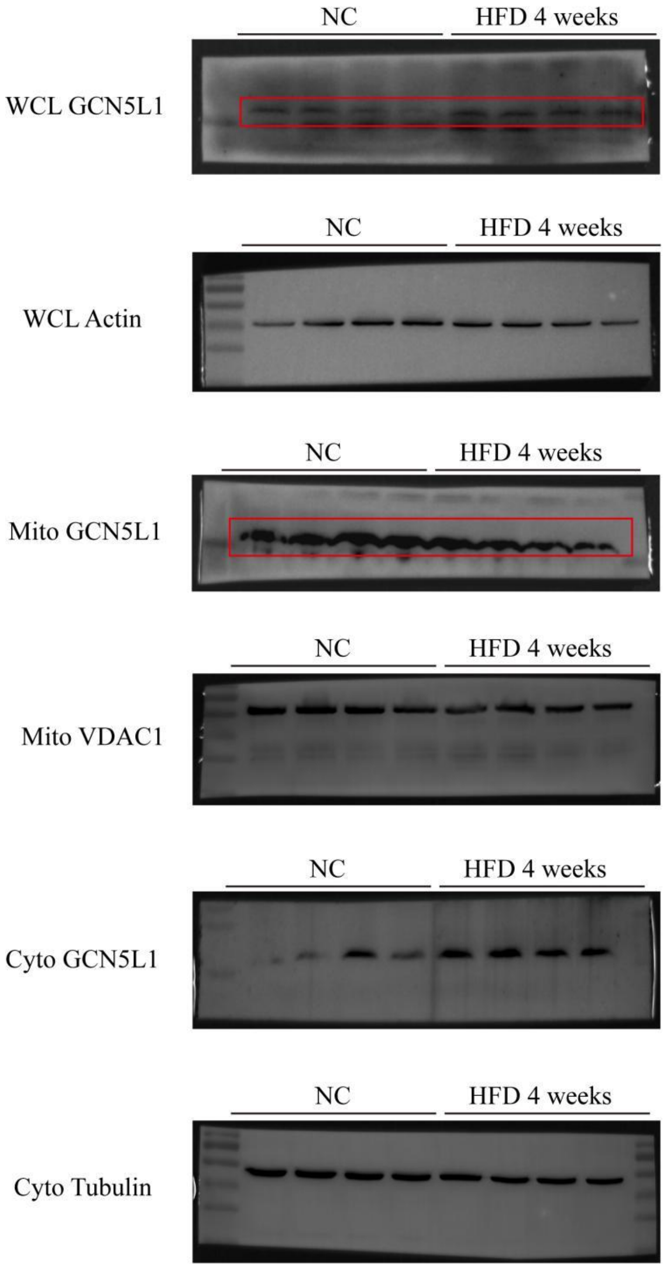

Full unedited gel for Figure 1I

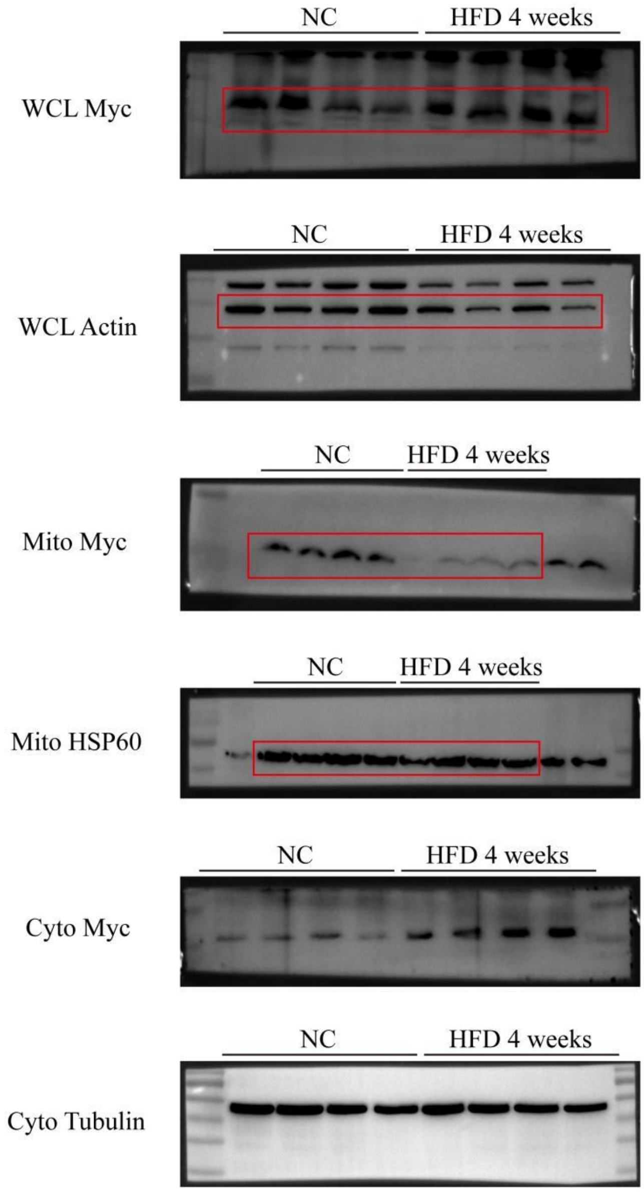

Full unedited gel for Figure 2G

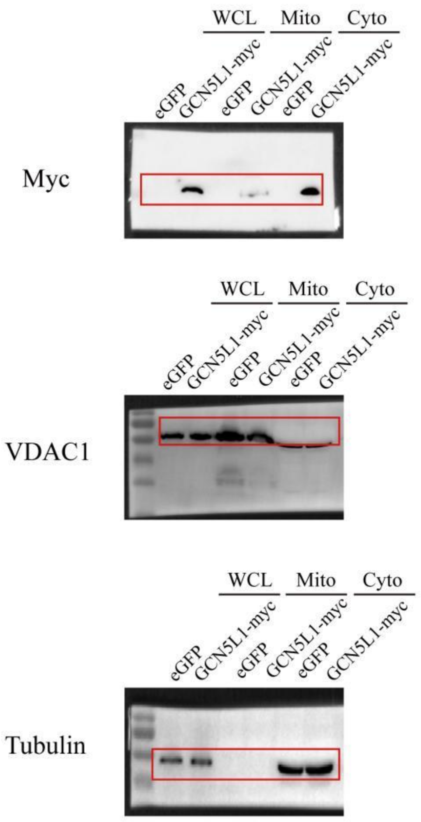

Full unedited gel for Figure 4E

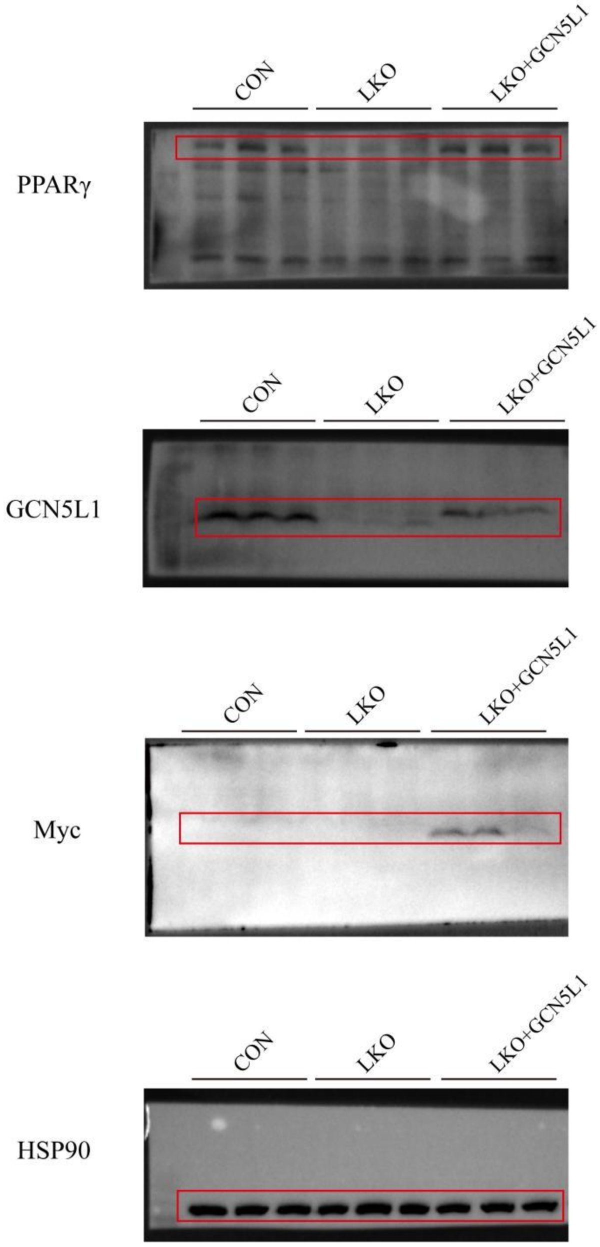

Full unedited gel for Figure 5A

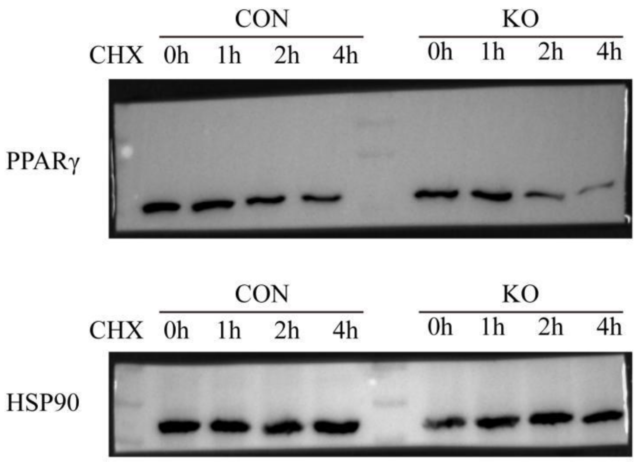

Full unedited gel for Figure 5B

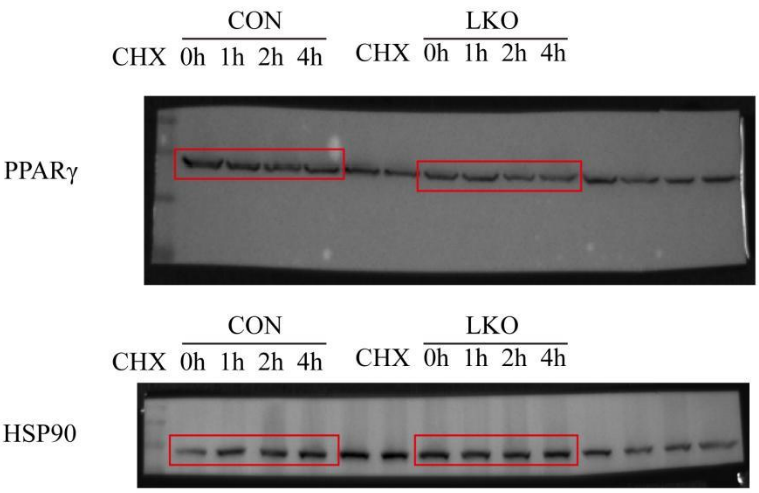

Full unedited gel for Figure 5C

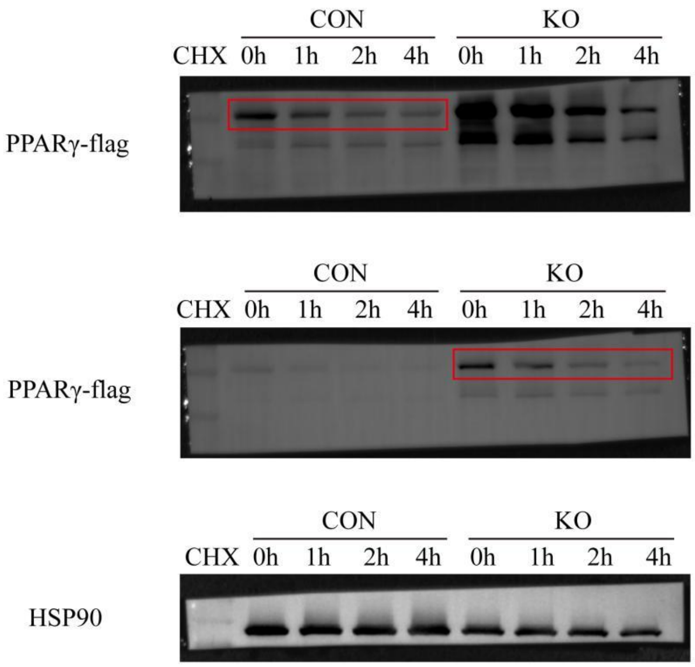

Full unedited gel for Figure 5D

MG132 Bafilomycin
CON KO CON KO CON KO

PPARγ

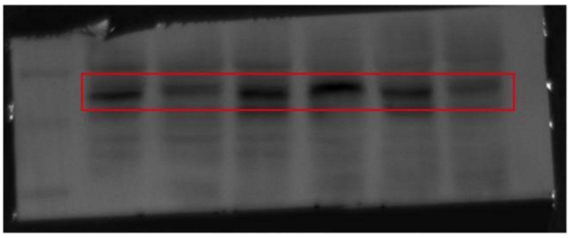

MG132 Bafilomycin
CON KO CON KO CON KO

HSP90

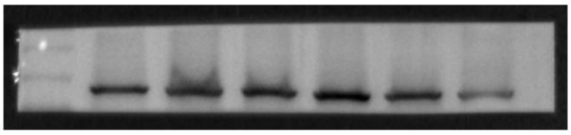

Full unedited gel for Figure 5E

CON KO

Ub-myc

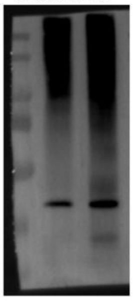

CON KO

ip: PPARγ-flag

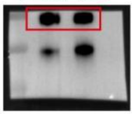

CON KO

input: PPARγ-flag

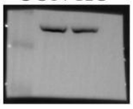

CON KO

HSP90

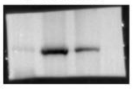

Full unedited gel for Figure 5F

CON KO

Ub-myc

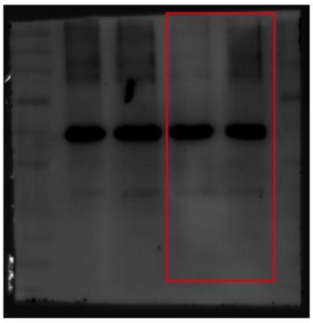

CON KO

ip: PPARγ-flag

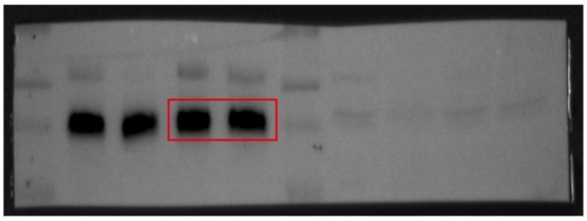

CON KO

input: PPARγ-flag

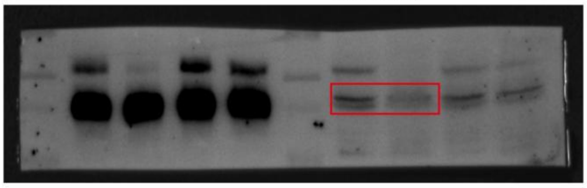

CON KO

input: HSP90

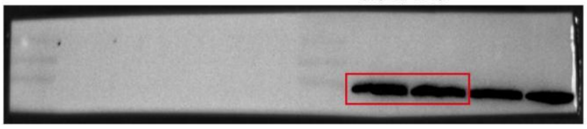

Full unedited gel for Figure 5G

CON KO KO+GCN5L1

Ub-myc

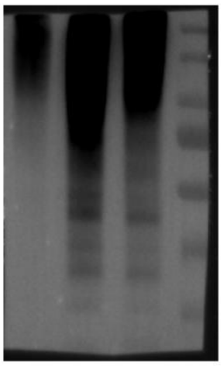

CON KO KO+GCN5L1

ip: PPARγ-flag

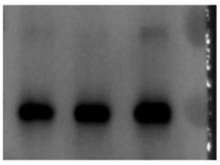

CON KO KO+GCN5L1

input: PPARγ-flag

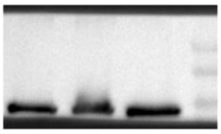

CON KO KO+GCN5L1

HSP90

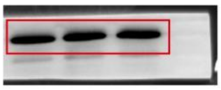

Full unedited gel for Figure 6A

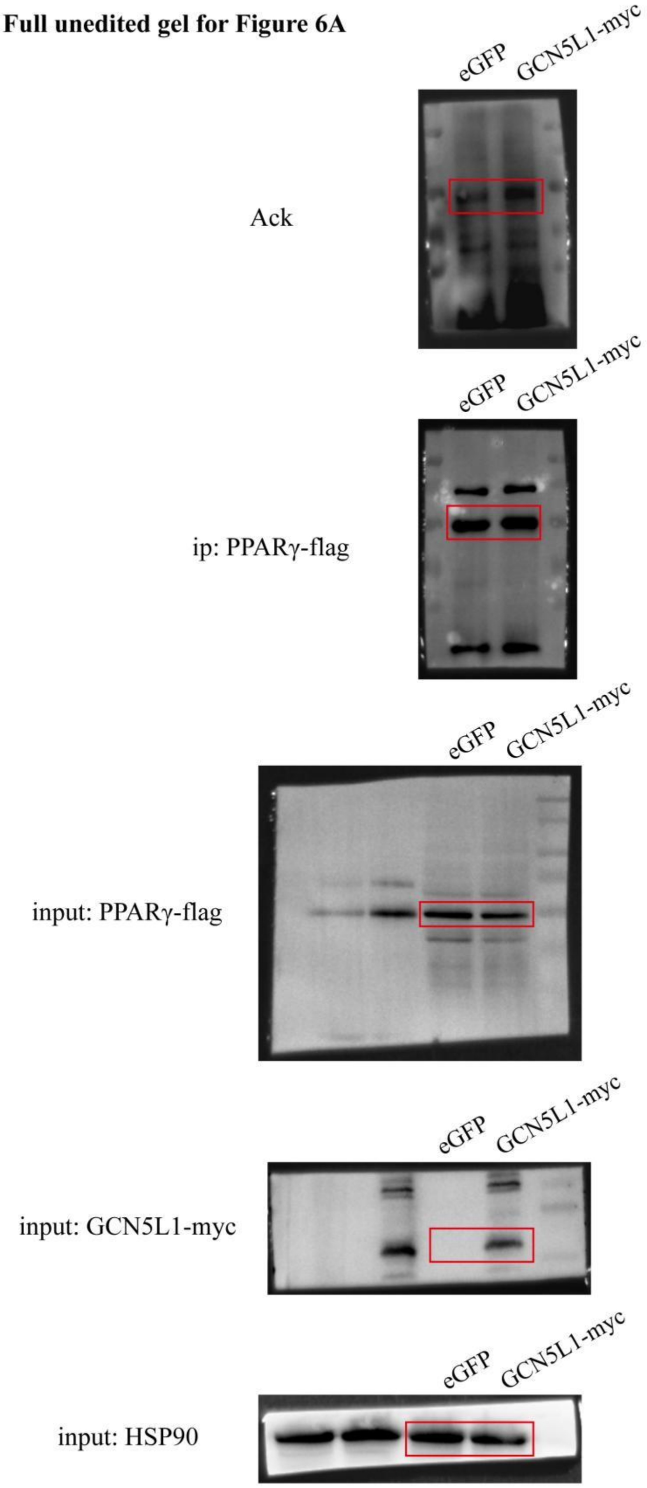

Full unedited gel for Figure 6B

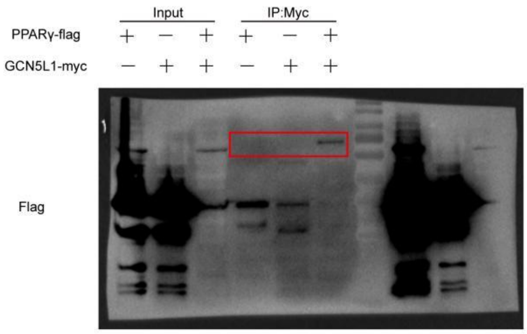

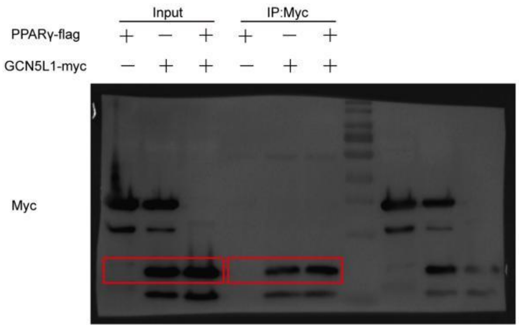

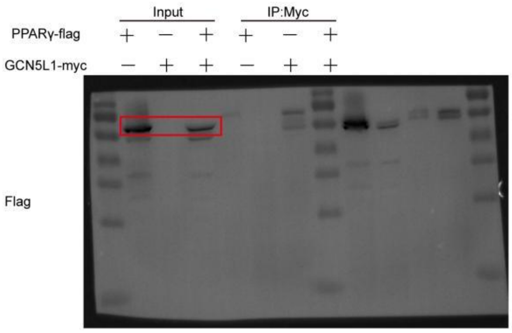

Full unedited gel for Figure 6C

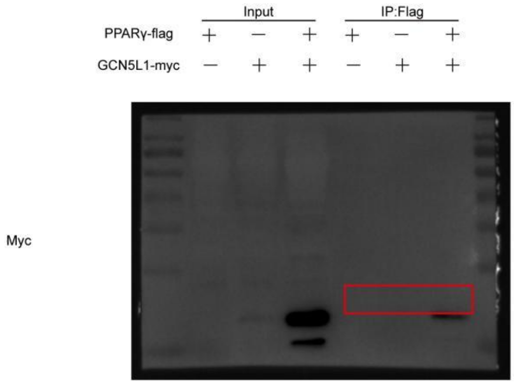

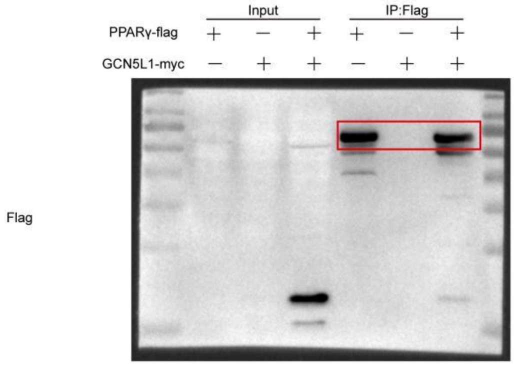

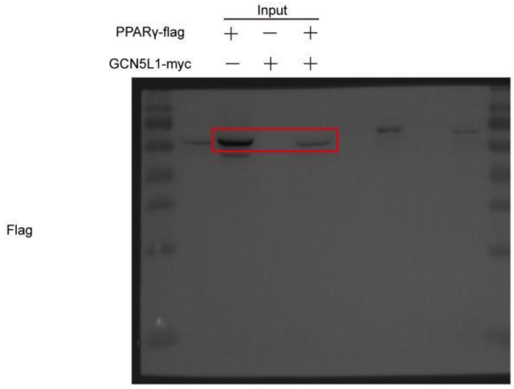

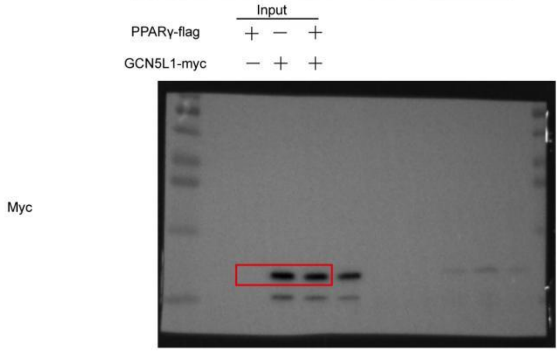

Full unedited gel for Figure 6D

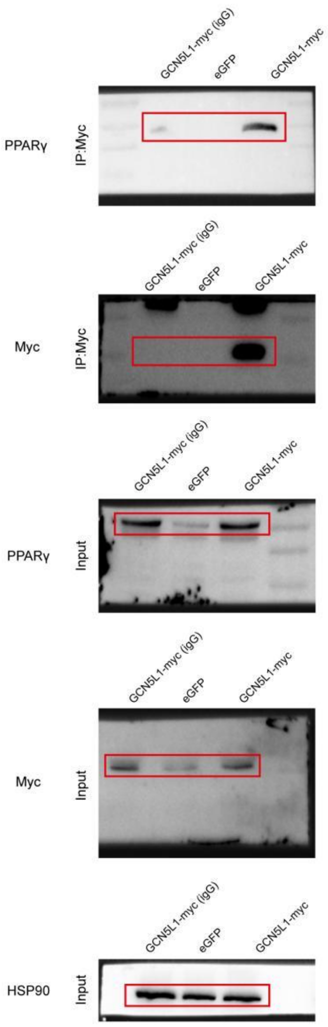

Full unedited gel for Figure 6F

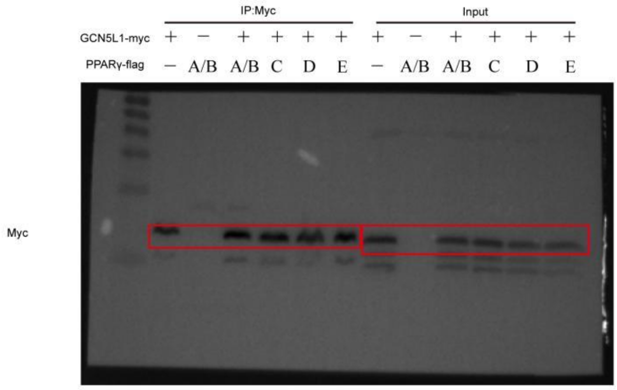

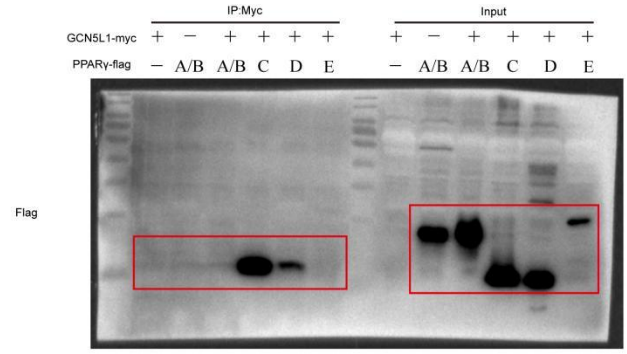

Full unedited gel for Figure 6I

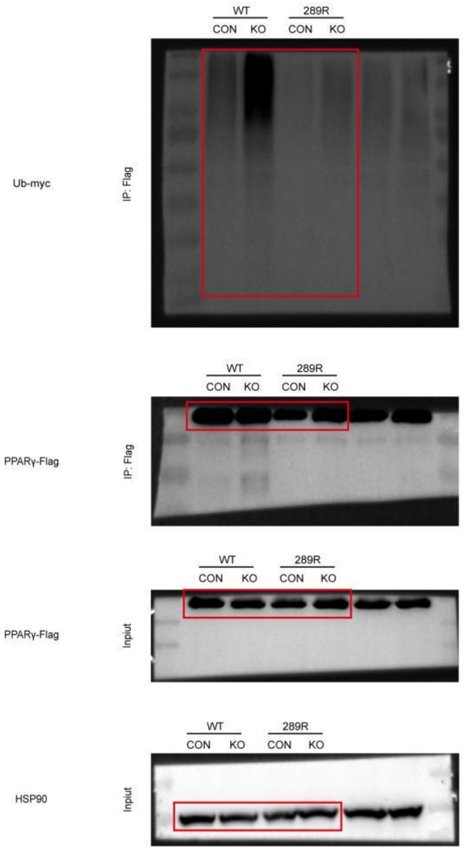

Full unedited gel for Figure 6J

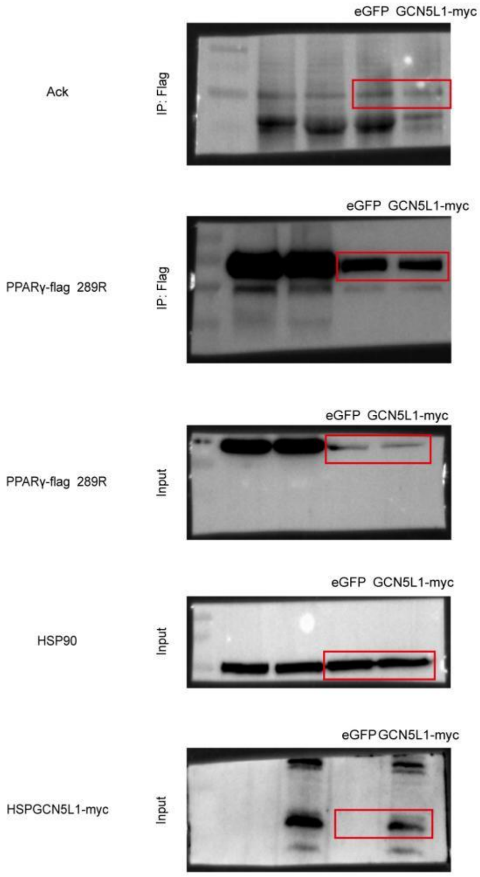

Full unedited gel for Figure 7E

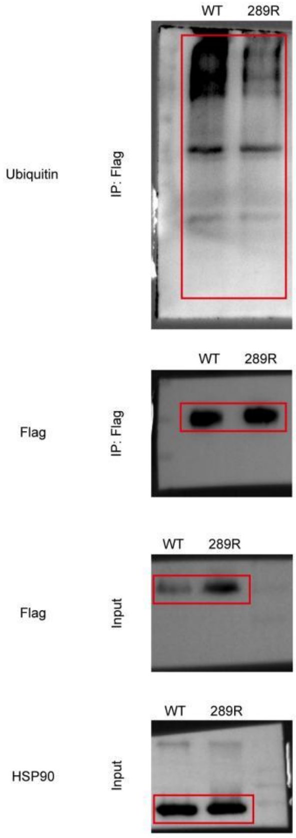

Full unedited gel for Figure S1B

Normal MASLD

Ack

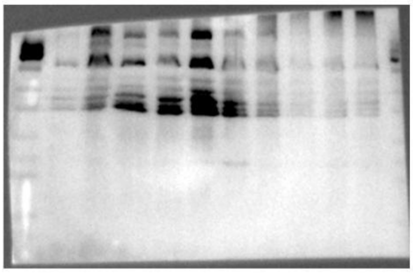

Normal MASLD

HSP60

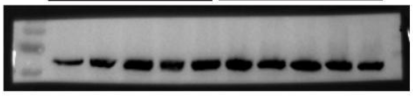

Full unedited gel for Figure S1C

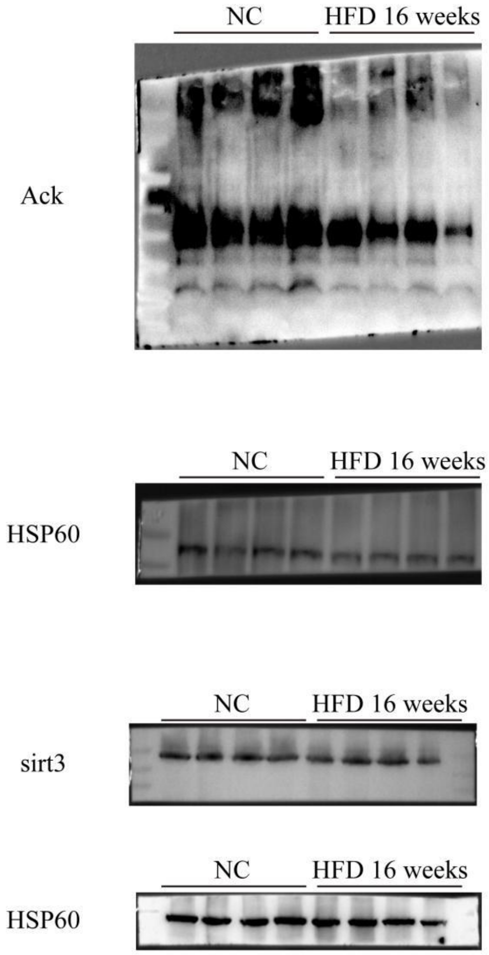

Full unedited gel for Figure S1D

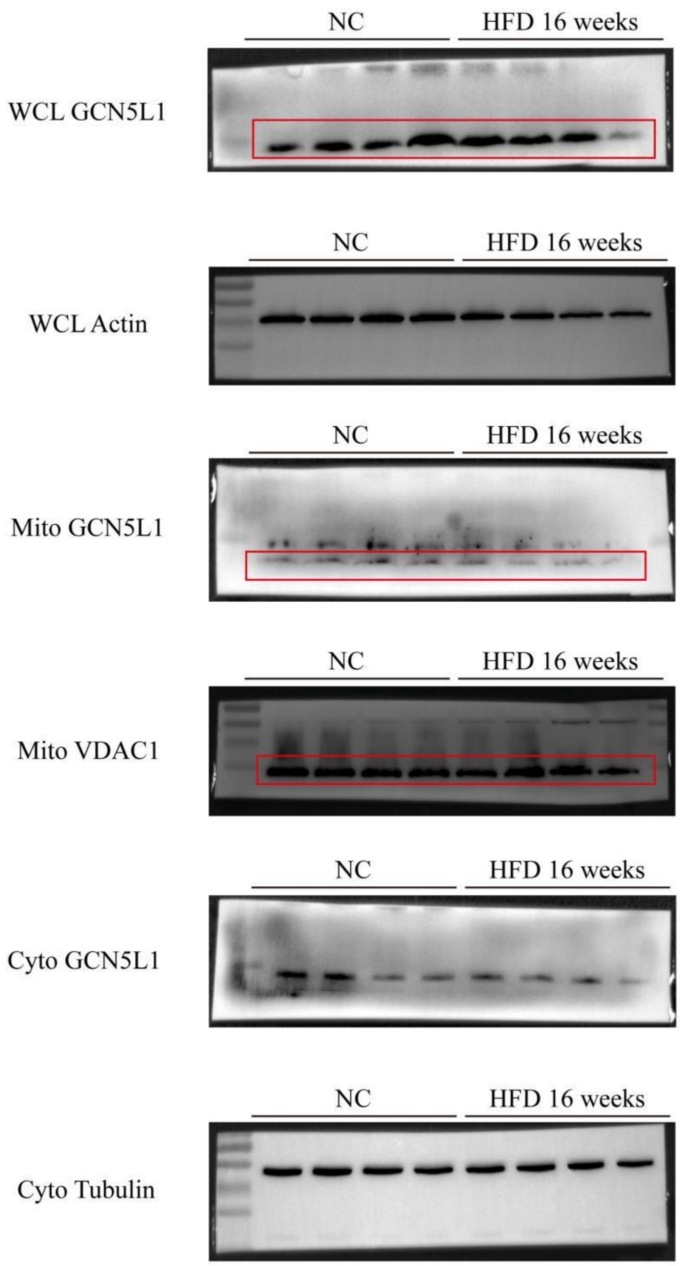

Full unedited gel for Figure S1G

WCL GCN5L1

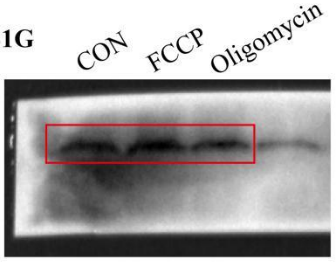

WCL Actin

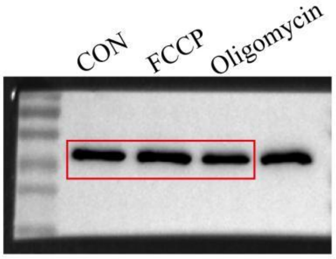

Mito GCN5L1

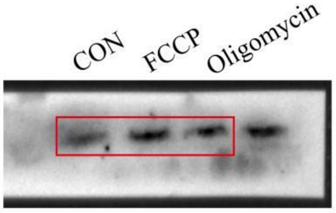

Mito VDAC1

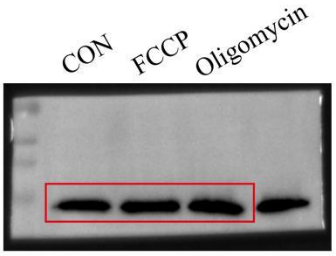

Cyto GCN5L1

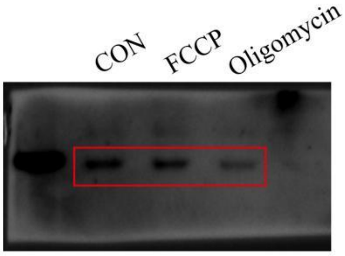

Cyto Tubulin

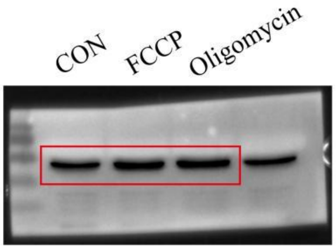

Full unedited gel for Figure S1H

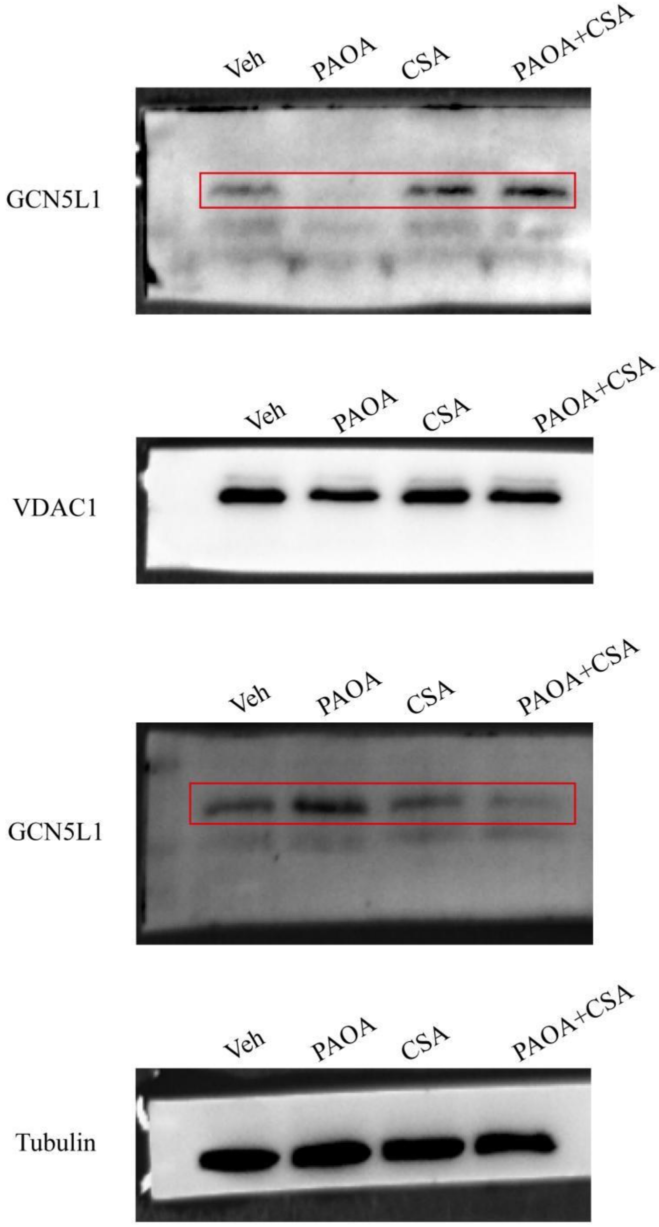

Full unedited gel for Figure S2L

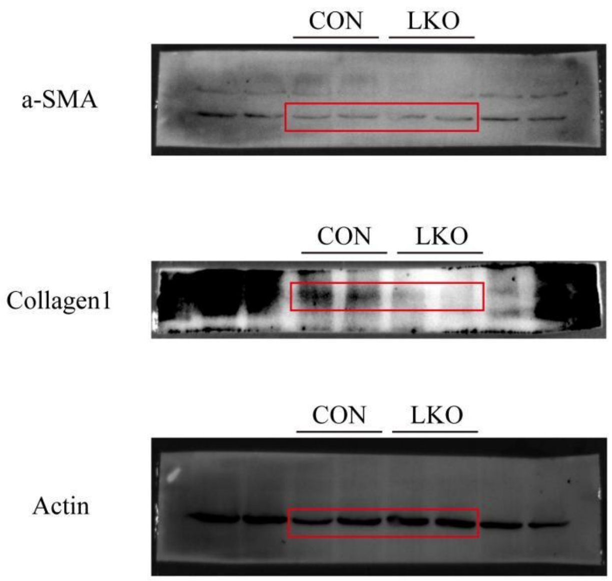

Full unedited gel for Figure S3E

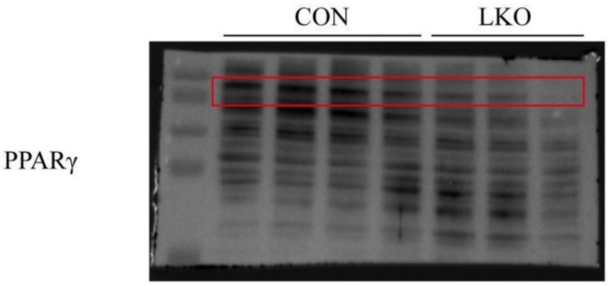

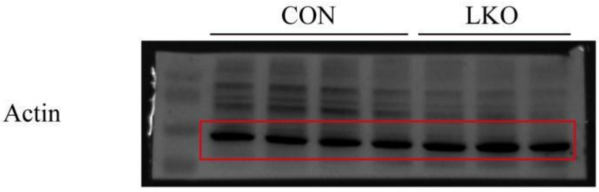

Full unedited gel for Figure S4A

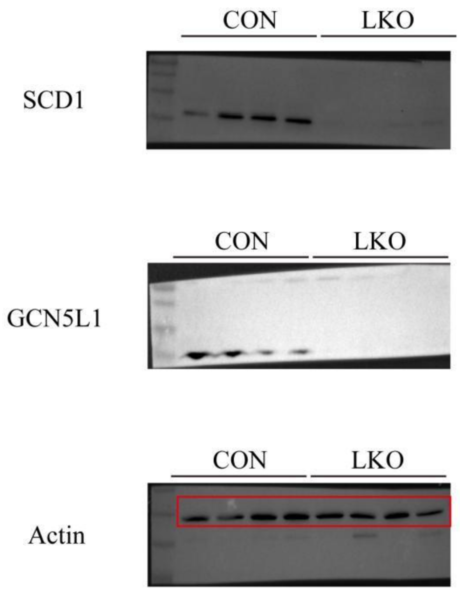

Full unedited gel for Figure S4B

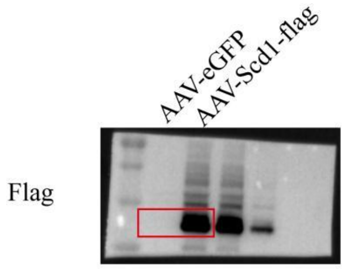

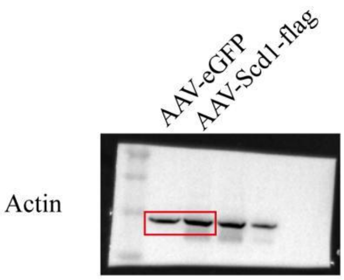

Full unedited gel for Figure S5A

CON KO

PPARγ

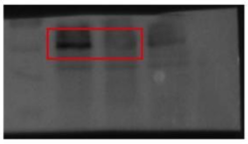

CON KO

Actin

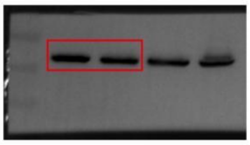

Full unedited gel for Figure S5B

CON KO

Ubiquitin

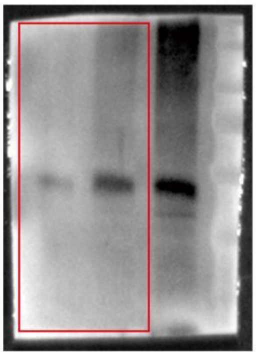

CON KO

ip: PPARγ

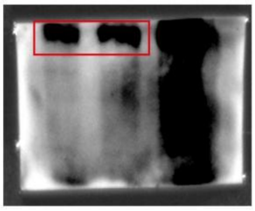

CON KO

input: PPARγ

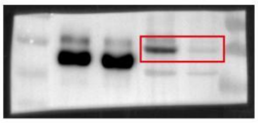

CON KO

input: HSP90

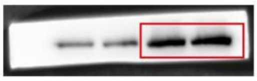

Full unedited gel for Figure S6A

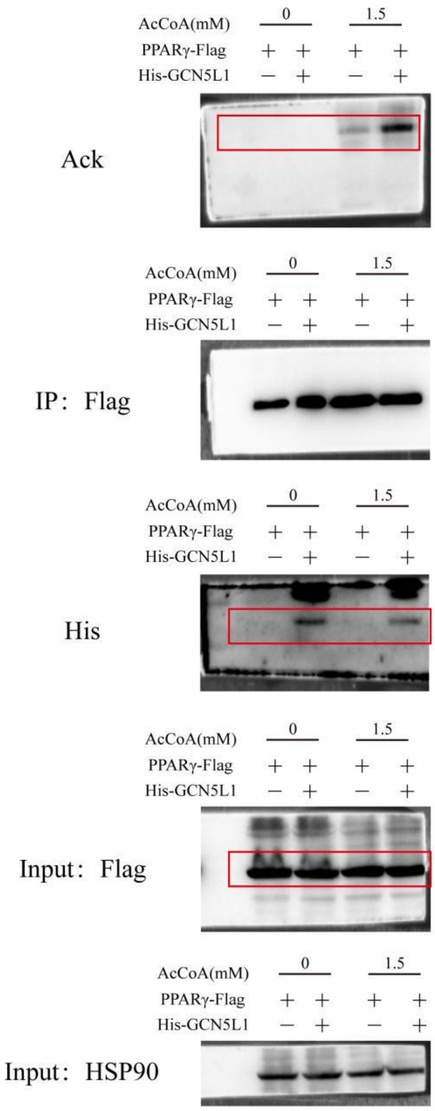

Full unedited gel for Figure S7A

eGFP WT 289R

Flag

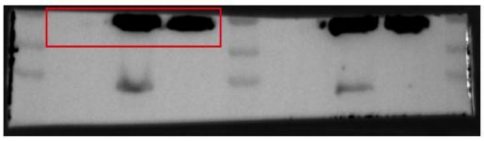

eGFP WT 289R

HSP90

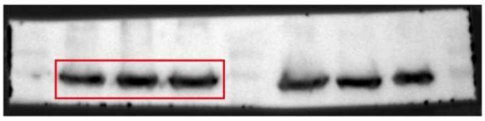

Supplement: Unedited blot and gel images [file jciinsight-11-196695-s060.pdf]
